# Cisplatin resistance can be curtailed by blunting Bnip3-mediated mitochondrial autophagy

**DOI:** 10.1038/s41419-022-04741-9

**Published:** 2022-04-22

**Authors:** Caterina Vianello, Veronica Cocetta, Daniela Catanzaro, Gerald W Dorn, Angelo De Milito, Flavio Rizzolio, Vincenzo Canzonieri, Erika Cecchin, Rossana Roncato, Giuseppe Toffoli, Vincenzo Quagliariello, Annabella Di Mauro, Simona Losito, Nicola Maurea, Cono Scaffa, Gabriele Sales, Luca Scorrano, Marta Giacomello, Monica Montopoli

**Affiliations:** 1 Department of Pharmaceutical and Pharmacological Sciences, University of Padova, Largo E. Meneghetti 2, 35131 Padova, Italy; 2 Department of Biology, University of Padova, Via Ugo Bassi 58B, 35131 Padova, Italy; 3 Center for Pharmacogenomics, Department of Internal Medicine, Washington University School of Medicine, 660 S. Euclid Ave., St. Louis, MO 63110 USA; 4 Sprint Bioscience, Huddinge, Sweden; 5 Department of Oncology-Pathology, Karolinska Institute, Stockholm, Sweden; 6 Department of Molecular Sciences and Nanosystems, Ca’ Foscari University of Venice, 30172 Venice, Italy; 7 Pathology Unit, Centro di Riferimento Oncologico di Aviano (C.R.O.) IRCCS, 33081 Aviano, Italy; 8 Department of Medical, Surgical and Health Sciences, University of Trieste, 34149 Trieste, Italy; 9 Experimental and Clinical Pharmacology Unit, Centro di Riferimento Oncologico (CRO), IRCCS, 33081 Aviano, Italy; 10 Division of Cardiology, Istituto Nazionale Tumori-IRCCS-Fondazione G. Pascale, Naples, Italy; 11 Pathology Unit, Istituto Nazionale Tumori-IRCCS-Fondazione G. Pascale, Naples, Italy; 12 Gynecologic Oncology, Istituto Nazionale Tumori-IRCCS-Fondazione G. Pascale, Naples, Italy; 13 Veneto Institute of Molecular Medicine, Via Orus 2, 35129 Padova, Italy; 14 Department of Biomedical Sciences, Via Ugo Bassi 58B, 35131 Padova, Italy

**Keywords:** Cancer therapeutic resistance, Mitophagy

## Abstract

Cisplatin (CDDP) is commonly used to treat a multitude of tumors including sarcomas, ovarian and cervical cancers. Despite recent investigations allowed to improve chemotherapy effectiveness, the molecular mechanisms underlying the development of CDDP resistance remain a major goal in cancer research. Here, we show that mitochondrial morphology and autophagy are altered in different CDDP resistant cancer cell lines. In CDDP resistant osteosarcoma and ovarian carcinoma, mitochondria are fragmented and closely juxtaposed to the endoplasmic reticulum; rates of mitophagy are also increased. Specifically, levels of the mitophagy receptor BNIP3 are higher both in resistant cells and in ovarian cancer patient samples resistant to platinum-based treatments. Genetic BNIP3 silencing or pharmacological inhibition of autophagosome formation re-sensitizes these cells to CDDP. Our study identifies inhibition of BNIP3-driven mitophagy as a potential therapeutic strategy to counteract CDDP resistance in ovarian carcinoma and osteosarcoma.

## BNIP3-mediated mitochondrial clearance as a mechanism underlying resistance to cisplatin

CDDP induces the stabilization of HIF-1α that leads to the expression of its target gene BNIP3. BNIP3 promotes autophagy of mitochondria by increasing their fission and degradation rates. This process ultimately boosts the ability of resistant cancer cells to escape CDDP cytotoxicity by eliminating one of its main targets, mitochondria, from which cytochrome c is released to initiate cell death. While BNIP3 expression could be a new prognostic factor to predict the chemotherapy responsiveness of cancer patients, targeting mitophagy could be a new therapeutic approach to overcome platinum resistance. We used BioRender Software (BioRender.com) to draw the schematic picture.

## Graphical abstract

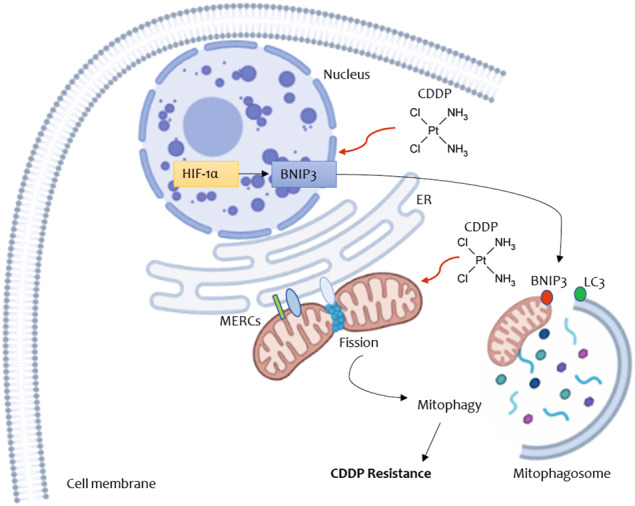

## Introduction

Cisplatin (CDDP) and platinum-based therapies are the frontline chemotherapeutic agents for the treatment of several solid tumors, including sarcoma, lung and ovarian carcinoma [1]. However, their clinical efficacy is limited due to the emergence of drug resistance, a multifactorial process whose molecular mechanisms are not completely understood. CDDP exerts its anticancer action by the formation of nuclear DNA adducts that activate the DNA damage response, trigger the proliferative arrest and hence the mitochondrial pathway of apoptosis, orchestrated by the BH3-only proteins BIM and PUMA [2]. Therefore, changes in the ability of mitochondria to respond to apoptosis might represent one of the mechanisms by which tumor cells acquire chemoresistance to CDDP. Activation of BAK and BAX, key regulators of mitochondria-dependent apoptosis belonging to the family of Bcl-2 proteins, is also involved in CDDP resistance, likely by overcoming the stimulation of mitochondrial outer membrane permeability (MOMP) and cytochrome c release that have been widely associated with CDDP cytotoxic effects [3–5]. Accordingly, Bcl-2 inhibitors synergize with cisplatin in the treatment of some types of cancer (e.g. non-small cell lung cancer, triple negative breast cancer and leukemia cells) [6–8]. Even though these findings highlight a central role of mitochondria in CDDP resistance, their contribution to this phenomenon is yet not clear.

Mitochondria-dependent apoptosis is counterbalanced by their selective degradation via autophagy (a process known as mitophagy) [9]. The relationship between autophagy and apoptosis is multifaceted due to the dual role of autophagy in tumor survival or cell death. In particular, cancer cells frequently exploit autophagy in order to escape to chemotherapeutics cytotoxicity and other stressful conditions within tumor such as hypoxia, nutrient deprivation or oxidative stress [10]. Hence, changes in mitophagy might represent another mechanism by which tumor cells acquire chemoresistance to CDDP. Mitophagy is inhibited during physiological T cell apoptosis to grant mitochondrial apoptosis execution. Indeed, inhibition of autophagosome biogenesis downstream of T cell receptor (TCR) activation leaves dysfunctional mitochondria untouched and free to release cytochrome c in the cytosol to activate effector caspases and execute TCR mediated apoptosis [11]. The opposite, i.e. enhanced mitophagy, can occur in cancer cells where an increase of mitochondrial clearance promotes chemoresistance by fostering the removal of their source of cytochrome c [12]. However, how mitophagy can counteract cytochrome c release and apoptosis, induced by drugs that classically engage the mitochondrial apoptotic pathway, is still unclear. Interestingly, mitochondrial fragmentation is a prerequisite for efficient mitophagy [13–15] and autophagosomes can form at the interface between the endoplasmic reticulum and mitochondria [16]. These contact points mark sites of mitochondrial fission and might therefore represent a hub where two reactions essential to clear mitochondria during chemoresistance occur: autophagosome biogenesis and mitochondrial fission. However, their potential role in chemoresistance has not been investigated. With these questions in mind, we capitalized on cellular models of cancers resistant to CDDP to verify if it could be ascribed to changes in mitochondrial morphology, contacts with the ER and BNIP3-driven mitophagy. BCL2/adenovirus E1B 19 kDa interacting protein 3 (BNIP3) is a proapoptotic Bcl-2 subfamily member localized in the Outer Mitochondria Membrane (OMM) [17]. At the mitochondria, BNIP3 can both inhibit the function of anti-apoptotic Bcl-2 proteins and trigger the mitophagic pathway [17–19]. BNIP3 as well as p62/SQSTM1 (Sequestosome-1) and AMBRA1 (Autophagy and Beclin 1 Regulator 1) are OMM-localized autophagy receptors (mitophagy receptors) able to attach autophagosomes to the OMM via their LC3 interacting region (LIR) motif. These factors target mitochondria to different clearance pathways regulated by various transcriptions factors (e.i. hypoxia and ubiquitination) [20, 21]. Many reports correlated high expression levels of BNIP3 with the aggressive cancer behavior in different tumor types like breast [22], colorectal [23], prostate [24] and endometrial [25]. Conversely, BNIP3 loss has been shown to correlate with bad prognosis in pancreatic cancer [26]. Also, other authors showed that BNIP3 loss increased angiogenesis, promoted tumor growth and breast cancer metastatization, due to the accumulation of dysfunctional mitochondria [27]. These in vivo studies provided contradictory evidence on the regulatory effects of BNIP3 in different cancer types, likely due to the co-participation of this mitophagy receptor in other signaling pathways. Further, BNIP3 has never been related to the effectiveness of chemotherapy response, whose molecular hallmarks remain a major goal in cancer research. To elucidate the mechanisms that confer resistance, we verified the expression of the mitophagic factor BNIP3 in both in vitro CDDP resistant models and cancer patients’ samples resistant to platinum-based chemotherapy, resulting in higher BNIP3 levels and enhanced mitophagy. Our overall data indicate that by reverting the excess mitochondrial autophagy we could curtail the resistance to CDDP observed in ovarian cancer and osteosarcoma, and that BNIP3 could be a potential target useful to overcome the resistance phenomena.

## Results

### In cisplatin-resistant cells, mitochondria are more fragmented and closer to the ER

**Fig. 1:**
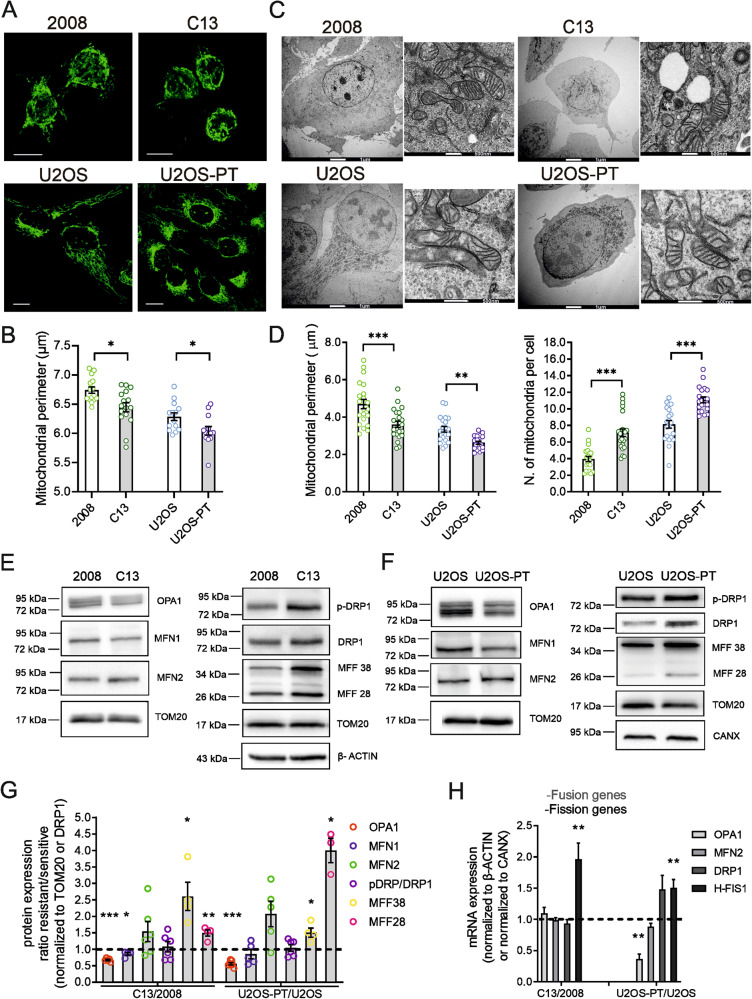
Mitochondria are fragmented in CDDP resistant cells. **A** Images of mitochondrial network in sensitive (2008, U2OS) and resistant (C13, U2OS-PT) cells acquired by confocal microscopy Zeiss using TOM20 immunostaining, 488. Scale bar 20 µm. **B** Mitochondria segmentation was performed using the ImageJ Squassh plugin (Rizk et al., 2014); size and morphology features were measured by using Fiji. Data from 15 different cells per cell line. *p < 0.05, calculated by a two-tailed unpaired t-test comparing resistant *vs* sensitive cells. **C** Images of mitochondrial morphology in sensitive (2008, U2OS) and resistant (C13, U2OS-PT) cells acquired by Tecnai G2 (FEI) transmission electron microscope operating at 100 kV; images were collected by a F114 (TVIPS) CCD camera. The TEM images and experiment are performed by the University of Padova electron microscopy facility. Scale bar 1 µm and 500 nm. **D** The morphometric analysis was performed using ImageJ freehand tool (at least 5 cells per sample, at least 50 images/sample). Data are the mean ± SEM of three different experiments; **p < 0.01, ***p < 0.001, calculated by a two-tailed unpaired t-test comparing resistant *vs* sensitive cells. **E**, **F** Expression of OPA1, MFN1, MFN2, MFFs, p-DRP1 and total DRP1. **G** The optical density (O.D.) was normalized respectively to TOM20; for total DRP1 to β-ACTIN for 2008-C13 or calnexin for U2OS-U2OS-PT in cancer cells sensitive and resistant. The data are expressed as ratio of resistant cells to sensitive. Data are the mean ± SEM of 4–5 different experiments; *p < 0.05; **p < 0.01, ***p < 0.001, calculated by a two-tailed unpaired *t*-test comparing resistant *vs* sensitive cells. **H** mRNA expression of genes OPA1, MFN2, DRP1 and H-FIS1 normalized to β-actin for 2008-C13 or calnexin for U2OS-U2OS-PT. The data are expressed as a ratio of resistant cells to sensitive cells set to 1. Data are the mean ± SEM of 4–5 different experiments; *p < 0.05; **p < 0.01, calculated by a two-tailed unpaired t-test comparing resistant *vs* sensitive cells.

**Fig. 2:**
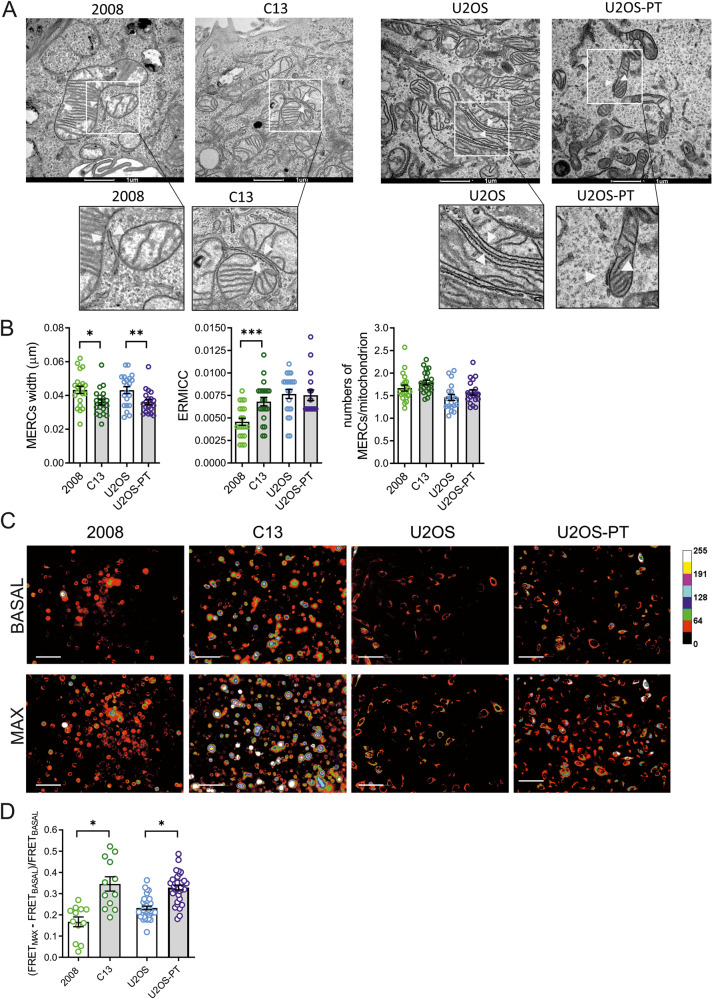
Resistant cells show an increased Mitochondria–ER proximity. **A** Images of mitochondria–ER contact sites in sensitive cells (2008, U2OS) and resistant (C13, U2OS-PT) acquired by Tecnai G2 (FEI) transmission electron microscope operating at 100 kV; images were collected by a F114 (TVIPS) CCD camera and the respective magnification of mitochondria–ER contact sites images. The TEM images and experiments are performed by the University of Padova electron microscopy facility. Scale bar 1 µm. **B)** The morphometric analysis of electron micrographs was performed using ImageJ freehand tool (7 cells per sample, at least 30 images/sample). We used the ER–mitochondria contact coefficient (ERMICC) to measure the extent of physical interaction among mitochondria and ER (as described in the Experimental procedures section). Data are the mean ± SEM of 3 different experiments; *p < 0.05, calculated by a two-tailed unpaired t-test comparing resistant *vs* sensitive cells. **C** Images of FRET signal using a modified FRET-based indicator of ER–mitochondria proximity (FEMP). **D** The respective measure of FRET signal of 2008-C13 and U2OS-U2OS-PT and quantification of the maximum MERC index for the indicated cell lines infected with Adenovirus FEMP. Scale bar 100 µm. Data represents mean ± SEM of 3 independent experiments; *p < 0.05, calculated by a two-tailed unpaired t-test comparing resistant *vs* sensitive cells. Cells from the raw images were segmented using YFP channel and intensity measures of CFP, YFP and FRET along with corresponding background intensities were calculated. Mean FRET Ratio intensity was then calculated by subtracting the background and normalizing to CFP intensity.

We set out to investigate the role of mitochondria shape in resistance to CDDP. To this end, we capitalized on two cell lines, 2008 (ovarian carcinoma) and U2OS (osteosarcoma), and their CDDP-resistant isogenic derivative cell lines (respectively, C13 and U2OS-PT), previously established in our laboratory [28], (Fig. S1A–C). At variance of the resistant ovarian cancer cells, the osteosarcoma cells didn’t show differences compared to their sensitive counterparts, in terms of mitochondrial functionality and mitochondrial membrane potential in basal condition (Fig. S1). Thus, we focused on mitochondrial morphology: the mitochondria appeared more fragmented in C13 and U2OS-PT cells (CDDP resistant) than in 2008 and U2OS cells (Fig. 1A, B). Electron micrographs confirmed that mitochondria were smaller in CDDP-resistant cells (Fig. 1C) and indeed, a morphometric analysis indicated a decrease in the mitochondrial perimeter (Fig. 1D). Because mitochondrial morphology is the result of the balance between fission and fusion reactions [29], we measured mRNA and protein levels of key players of these processes (Fig. 1E–H). Resistant cells expressed higher levels of fission proteins (h-Fis1 mRNA and MFFs protein in C13; h-Fis1 and DRP1 mRNA and MFFs protein in U2OS-Pt) and lower levels of the pro-fusion protein OPA1. Conversely, we found increased levels of the mitochondrial outer membrane fusion protein Mfn2. Because Mfn2 also tethers ER to mitochondria [30], we reasoned that its increased levels might be associated not to mitochondrial elongation, but to changes in the ER–mitochondria juxtaposition. We therefore turned back to the analysis of electron microscopy images of resistant cancer cells, where we measured indeed an increase of contacts between mitochondria and ER and a reduction in the distance between the two organelles (Fig. 2A, B). We corroborated these findings with an objective assay of mitochondria–ER proximity, employing a validated FRET based probe (FEMP) [29]. The normalized FRET value (that calculate the FRET signal before and after rapamycin treatment that induced forced FEMP dimerization) [31] was higher in resistant cells lines, confirming that mitochondria–ER proximity was increased in the CDDP-resistant cells analyzed here (Fig. 2C, D). Because activation of the ER stress response associated with CDDP resistance [32] can also increase ER–mitochondria proximity [33], we explored expression of GRP78 and ATF4, two key proteins that modulate ER stress. However, their levels were unchanged, suggesting that ER stress does not contribute to the increased juxtaposition measured in the resistant cell lines (Fig. S2).

Mitochondrial fragmentation can be an early hallmark of apoptosis: as we observed changes in the levels of mitochondrial fission proteins in resistant cells, we hypothesized that apoptotic signaling could be impaired in these models. Indeed, apoptosis reduction has been proposed as a key mechanism in the resistance process [34]. We verified this possibility by analyzing the expression levels of the Bcl-2 family members BAK and BID, that regulate permeability of the outer mitochondrial membrane. Our results suggest that the CDDP-resistant cells are not characterized by altered pro apoptotic signaling (Fig. S2).

### BNIP3 and mitophagy are increased in cisplatin-resistant cells and in ovarian cancers resistant to platinum-based chemotherapy

**Fig. 3:**
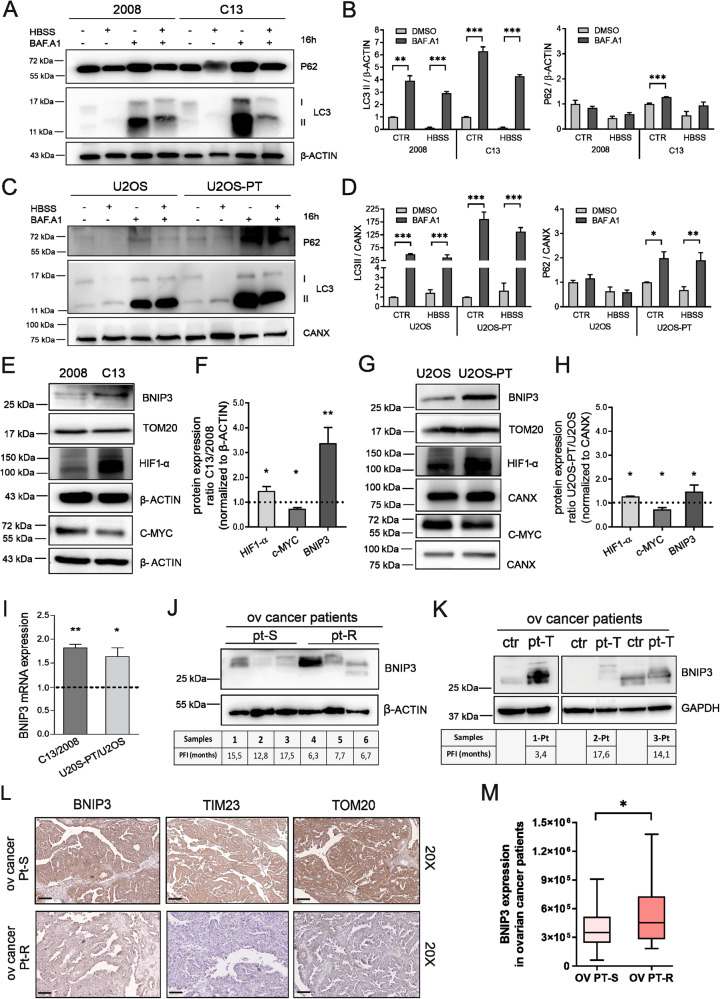
Resistant clones boost the autophagic flux in association with higher expression of hypoxia-induced BNIP3. **A** and **C** Autophagic flux was measured by assessing levels of p62, LC3 BI, LC3 BII after 16 h starvation in HBSS, upon 200 nM Bafilomycin A1 treatment or HBSS in combination with Bafilomycin A1. The optical density was normalized on β-ACTIN (2008-C13) (**B**) and (**D**) on calnexin (U2OS-U2OS-PT). The data are expressed in the percentage of treated cells with respect to untreated cells. Data are the mean ± SEM of 4–5 different experiments. *p < 0.05; **p < 0.01; ***p < 0.001, calculated by a two-tailed unpaired t-test comparing Bafilomycin A1 treatment *vs* DMSO treated cells. **E** BNIP3, c-MYC, HIF-1α protein expression of 2008 and C13 normalized respectively to TOM20 and to β-ACTIN and respective quantification (**F**). BNIP3, c-MYC, HIF-1α proteins expression of U2OS-U2OS-PT normalized respectively to TOM20 and to calnexin (**G**) and respective quantification (**H**). Data are expressed as the ratio of resistant cells to sensitive. Data are the mean ± SEM of 4–5 different experiments; *p < 0.05, **p < 0.01; calculated by a two-tailed unpaired t-test comparing resistant *vs* sensitive cells. **I** mRNA expression of BNIP3 gene normalized on β-ACTIN (2008-C13) and calnexin (U2OS and U2OS-PT). The data are expressed as a ratio of resistant cells to sensitive set to 1. *p < 0.05, **p < 0.01, calculated by a two-tailed unpaired t-test comparing resistant *vs* sensitive cells. **J** BNIP3 expression in ovarian cancer patients sensitive (pt-S) and resistant (pt-R) to platinum-based chemotherapy (platinum free interval, PFI, if lower than 12months has been defined to classify resistant patients). **K** BNIP3 expression in another ovarian cancer patients’ group before and after platinum-based chemotherapy (ctr refers to non-treated patient and pt-T to treated patient). The first patient was treated with carboplatin in combination with doxorubicin and presented a higher BNIP3 expression and lower survival (PFI < 12months). The other two patients were treated with carboplatin in combination with taxol and presented lower BNIP3 expression and higher survival (PFI > 12months). **L** Representative images of ovarian cancer specimens (*n* = 2) showing low (0–20%) and high (55–100%) staining of TIM23, TOM20 and of BNIP3 in tumor tissue. Black scale bars = 100 μm. Between the two cancer patients treated with platinum-based chemotherapy after surgery, lower TIM23 and TOM20 protein expression was associated with platinum-resistant patient (pt-R), as compared to platinum-sensitive patient (pt-S). **M** Among the 295 ovarian cancer patients treated with platinum-based therapy (cisplatin, carboplatin, or oxaliplatin after surgery), high BNIP3 protein expression was associated with lower PFS (OV PT-R, ovarian cancer patient resistant to platinum-therapy) compared to patients with low BNIP3 expression (OV PT-S, ovarian cancer patient sensitive to platinum-therapy) (Wilcox-rank test, two-sided, *p* < 0.05).

A feature prominent in the electron micrographs of the CDDP-resistant cells was the overall increase in the number of smaller mitochondria (Fig. 1D). Because fragmented mitochondria are usually targeted for degradation by autophagy, we thought that fragmented mitochondria accumulated in the CDDP-resistant clones because their autophagic removal was altered. We therefore measured levels of the bona fide autophagosome cargoes LC3 and p62 in fed and starved cells. As expected, starvation stimulated the accumulation of LC3-II and p62 in the presence of the vacuolar ATPase inhibitor Bafilomycin A1 that prevents autophagosomes acidification. The flux of autophagy, calculated by the ratio of the normalized LC3-II and p62 between Bafilomycin-treated and untreated cells, appeared increased in resistant cells (Fig. 3A–D). Considering our previous findings, we hypothesized that mitochondrial fragmentation could participate in cancer cell growth and tumor progression upon induction of CDDP resistance. In particular, mitochondrial fragmentation could help to segregate dysfunctional mitochondria and their removal through mitochondria-selective autophagy (the process named mitophagy).

One of the main mitophagic regulators is BNIP3, a receptor for the recruitment of autophagic machinery at the surface of mitochondria. With respect to the parental cells, C13 and U20S-PT resistant cells showed higher BNIP3 expression (Fig. 3E–I). In parallel, we measured BNIP3 expression in ovarian cancer patient samples. The platinum-free treatment interval (PFI) was defined as the interval between the end of the first-line platinum-based treatment and the date of first recurrence/progression as in Garziera et al. 2019 [35]. Higher levels of BNIP3 in samples isolated from ovarian cancer patients (treated with carboplatin together with doxorubicin or taxol) were associated with reduced survival, suggesting a role for BNIP3 in platinum-related resistance mechanism (Fig. 3J, K).

To further understand the contribution of BNIP3 in platinum-related mitochondrial autophagy, we also performed immunohistochemistry experiments in other samples of ovarian cancer patients (either sensitive or resistant). The weak staining intensity of the mitochondrial markers TIM 23 (Translocase of the Inner Membrane 23) and TOM20 (Translocase of the Outer Membrane 20) in resistant samples, with respect to the sensitive control, suggested that resistance is coupled to a lower mitochondrial mass caused by an increased mitophagy flux (Fig. 3L and Fig. S4A–C).

To further strengthen our conclusion, we performed a bioinformatic analysis using the RNA-seq dataset generated by the TCGA consortium. We focused our investigation on the ovarian carcinoma samples described in [36]. We set a threshold on the progression-free survival interval (PFS, as defined in [36]) at 9 months to separate patients into two groups: sensitive (207 individuals) or resistant (88 individuals) to platinum-based therapy. We could observe a significant difference in BNIP3 expression levels (two-sided Wilcoxon test, p-value: 0.0165): indeed the patients with lower PFS showed an increased expression of BNIP3 (Fig. 3M).

Notably, BNIP3 is a well-known HIF-1α target gene and its expression, together with the consequent mitophagy activation and inhibition of c-Myc-dependent mitochondrial biogenesis, promotes cancer progression and resistance to chemotherapy [37]. Thus, we decided to study the expression of these two nuclear transcription factors involved in metabolic reprogramming of cancer cells and our data showed that resistant cells are characterized by higher levels of HIF-1α and lower levels of c-MYC (Fig. 3E–H).

### BNIP3-driven mitophagy is involved in cisplatin resistance mechanism

We investigated the role of BNIP3-driven mitophagy in the CDDP resistance process. To address this point, we transiently downregulated the expression of BNIP3 both in sensitive and resistant cells and, in these models, we analyzed the mitochondrial mass and the mitophagy.

To estimate the mitochondrial mass, we analyzed first the levels of mitochondrial proteins located in different compartments: Cyclophilin D (CYD, enriched in the matrix), Complex IV (inner mitochondrial membrane) and TOM20 (Translocase of the outer membrane), in basal conditions and following starvation (Fig. S3A–D). We found that CDDP resistant clones are characterized by lower mitochondrial mass during starvation. In addition, CDDP treatment led to a significant increase in the expression levels of same mitochondrial proteins only in sensitive cells and not in resistant cells (Fig. S3E–H). Accordingly, the mitochondrial mass measured by MTG (mitotracker green) intensity increased significantly upon CDDP exposure only in sensitive but not in resistant cells (Fig. S3I).

**Fig. 4:**
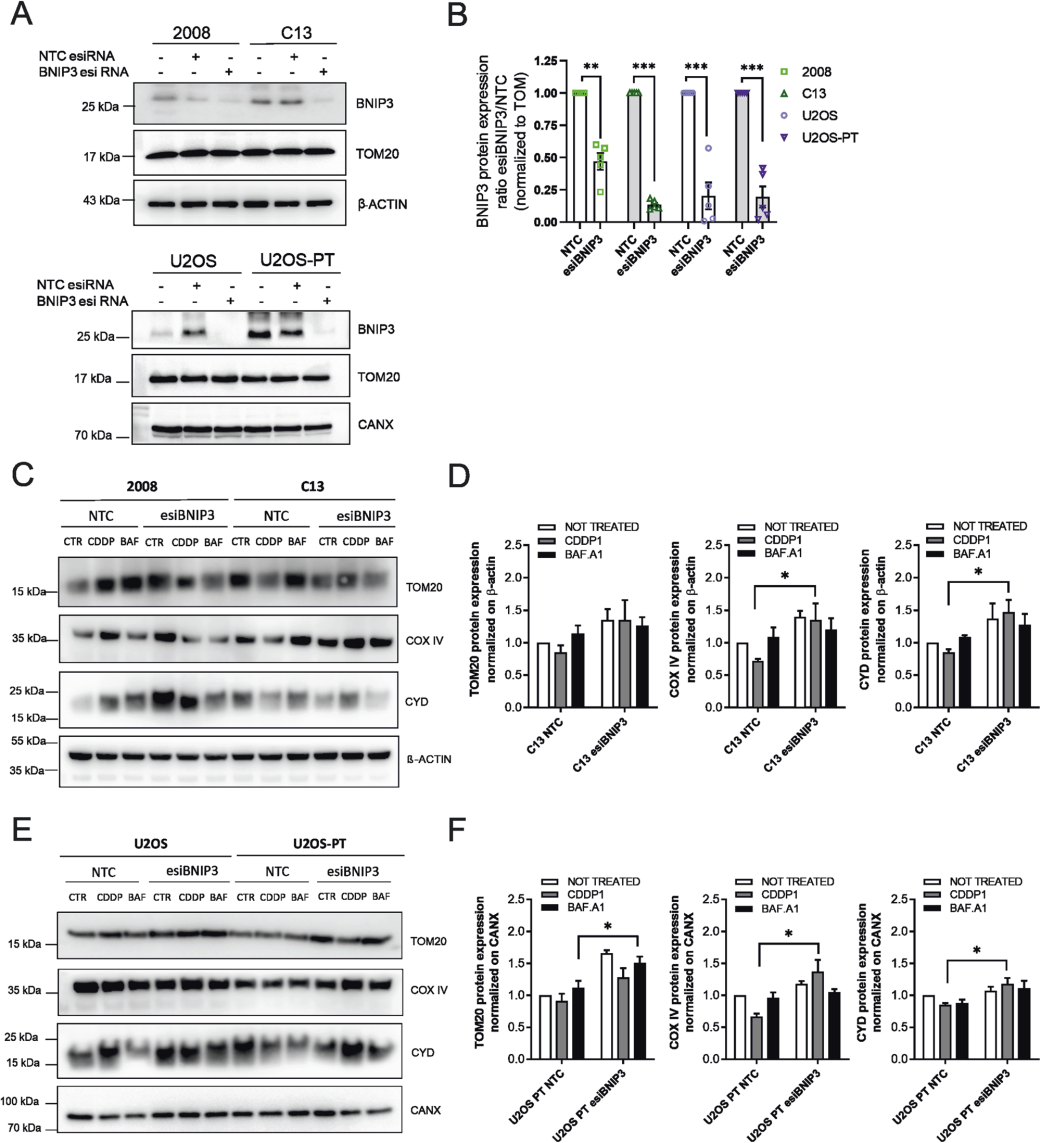
BNIP3 silencing reduces mitochondrial mass loss. **A** 2008-C13 and U2OS-U2OS-PT were transfected with a non-targeting control (NTC, scramble) esiRNA or with an esiRNA targeting BNIP3 (esiBNIP3). BNIP3 protein expression levels were normalized to TOM20; in (**B**) the respective quantification. Data are the mean ± SEM of 5 different experiments. **p < 0.01, ***p < 0.001, calculated by a two-tailed unpaired *t*-test comparing esiRNA targeting BNIP3 *vs* non-targeting control cells. (**C**) and (**E**) Effect of 24 h of CDDP (1 µM) and Bafilomycin A1 (100 nM) treatment on mitochondrial proteins expression of 2008-C13 and U2OS-U2OS-PT transfected with non-targeting control (NTC, scramble) esiRNA or with an esiRNA targeting BNIP3 (esiBNIP3), and the respective quantification (**D**) and (**F**). The optical density of TOM20, Cyclophilin D and COX IV was normalized to β-ACTIN for ovarian cancer cells and to calnexin for osteosarcoma cells sensitive and resistant. The data are expressed as the ratio of treated cells respect to untreated cells; *p < 0.05, calculated by a two-tailed unpaired t-test comparing esiRNA targeting BNIP3 (esiBNIP3) *vs* non-targeting control (NTC, scramble) cells. Data are the mean ± SEM of 4–5 different experiments.

When BNIP3 was downregulated in resistant cells, CDDP induced an increase in the expression of mitochondrial proteins as compared to non-targeting control cells. These findings suggested that BNIP3 knockdown in resistant cells can rescue the loss of mitochondrial mass after CDDP treatment (Fig. 4).

**Fig. 5:**
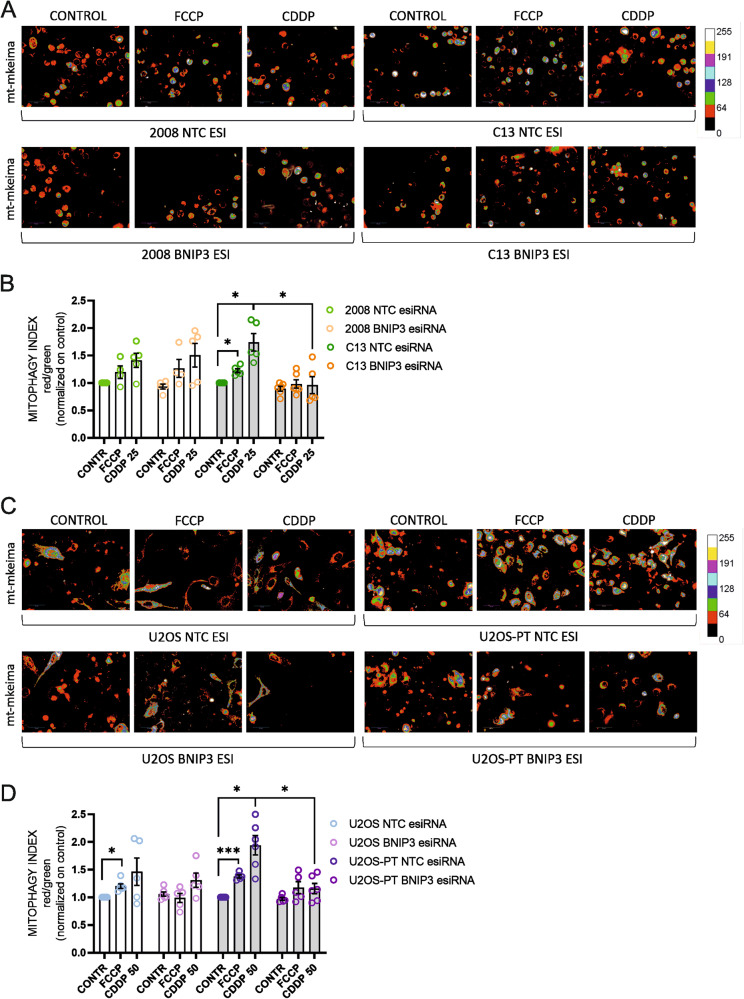
BNIP3 silencing suppresses mitophagy. Effect of 24 h of CDDP (25–50 µM) and of overnight of FCCP (10 µM) on mitophagy of 2008-C13 (**A**) and U2OS-U2OS-PT (**C**) transfected with non-targeting control (NTC, scramble) esiRNA or with an esiRNA targeting BNIP3 (esiBNIP3). The respective Mitophagy Index and quantification is reported in (**B**) and (**D**). Data are the mean ± SEM of 5–6 different experiments. *p < 0.05; calculated by a two-tailed unpaired t-test comparing esiRNA targeting BNIP3 (esiBNIP3) cells *vs* non-targeting control (NTC, scramble) esiRNA cells; *p < 0.05, ***p < 0.001, calculated by a two-tailed unpaired *t*-test comparing treated cells *vs* untreated cells.

Besides, we performed experiments with the mitochondria-targeted mitophagy indicator mt-Keima in High Content Imaging Settings. We measured the Mitophagic Index upon FCCP exposure, as a positive control (Fig. S5D), and CDDP treatment. Our results showed that BNIP3 silencing blocks mitophagy upon CDDP treatment in resistant but not in sensitive cells (Fig. 5). Then we performed immunoblotting for BNIP3, P62 and LC3 (as control for Bafilomycin inhibitory effect) to test the blocking of autophagy in our in vitro models (in the same experimental conditions used for the datasets summarized in Fig. 4A, B). We found a significant increase of p62 accumulation upon Bafilomycin treatment and an increased BNIP3 expression upon CDDP treatment only in resistant cells but not in their sensitive counterparts. All these effects were abolished when BNIP3 was downregulated (Fig. S5E–G).

It is well known that different mitophagy processes are modulated by different transcriptional responses: while the Parkin-PINK1 pathway is induced by mitochondrial or ER stress signals [38], BNIP3 expression is mostly driven by the hypoxia-inducible factor HIF-1α, as in the case of our in vitro CDDP resistant models [20]. Besides, it has been already shown that BNIP3 promotes clearance also of polarized mitochondria [12] and is responsible of the basal mitochondrial turnover, independently of the polarization status of these organelles [39].

We verified that other mitophagic pathways are not involved in CDDP resistance in our models, by analyzing the expression of PARKIN, PINK1 and AMBRA1 (Fig. S5A–C). The results of these experiments showed that expression of these mitophagy markers do not increase in CDDP resistant cells as compared to their sensitive counterparts.

These evidence confirm that BNIP3 specifically mediates the mitophagic process in CDDP resistant cells.

### Inhibition of mitochondrial autophagy re-sensitize cancer cells to cisplatin

The challenge in this work is to determine what selective advantage is conferred to the resistant cells through upregulation of mitophagy and to understand if mitophagy promotes cancer survival. We addressed this point through two different procedures: a genetic and a pharmacological approach.

**Fig. 6:**
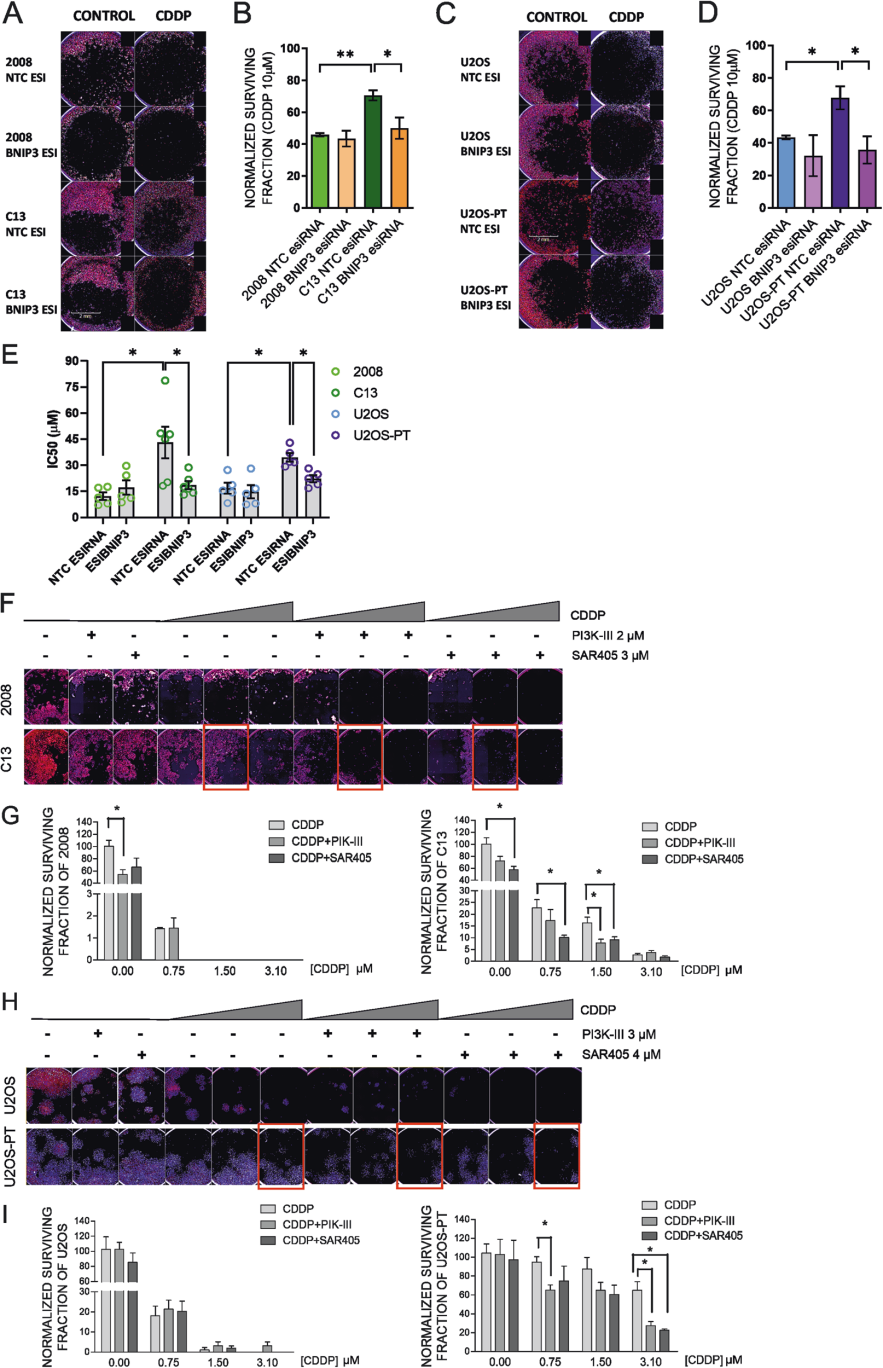
BNIP3 silencing and pharmacological inhibition of autophagy promotes CDDP sensitivity. Clonogenic assay of 2008-C13 (**A**) and U2OS-U2OS-PT (**C**) cells transfected with a control NTC esiRNA (scramble esiRNA) or esiRNA targeting BNIP3 (esiBNIP3) and treated for 24 h with CDDP (10–50 µM). The respective quantification of normalized surviving factor of the experiment in (**A**–**C**) is reported in (**B**) and (**D**). Data are the mean ± SEM of 4–5 different experiments. *p < 0.05; **p < 0.01; calculated by a two-tailed unpaired t-test comparing esiBNIP3 cells *vs* NTC esiRNA cells and comparing resistant *vs* sensitive counterparts. **E** IC50 of viability curves upon 24 h of CDDP (1–5–10–25–50 µM) of 2008-C13 and of U2OS-U2OS-PT transfected with non-targeting control (NTC, scramble) esiRNA or with an esiRNA targeting BNIP3 (esiBNIP3). Data are the mean ± SEM of 5–6 different experiments. *p < 0.05, calculated by a two-tailed unpaired t-test comparing esiRNA targeting BNIP3 (esiBNIP3) resistant *vs* sensitive cells and non-targeting control (NTC, scramble) esiRNA resistant *vs* sensitive cells; *p < 0.05, esiRNA targeting BNIP3 cells *vs* non-targeting control cells. Clonogenic assay of ovarian cancer cells treated for 72 h with CDDP (0.75–1.5–3.1 µM) in combination with either DMSO, 3 µM PIK-III or 2 µM SAR-405 (**F**). Quantification of the normalized surviving factor (**G**) of the experiment shown in (**F**). Clonogenic assay of osteosarcoma cells treated for 72 h with CDDP (0.75–1.5–3.1 µM) in combination with either DMSO, 3 µM PIK-III or 4 µM SAR-405 (**H**). Quantification of the normalized surviving factor (**I**) of the experiment shown in (**H**). Data are the mean ± SEM of 4–5 different experiments. *p < 0.05; calculated by a two-tailed unpaired *t*-test comparing combination treatment *vs* the CDDP treatment.

First, to point out the involvement of mitophagy in cisplatin resistance, we silenced BNIP3 and measured cell viability and survival upon CDDP treatment. Our data show that BNIP3 knockdown in resistant cells restores sensitivity to cisplatin both in viability and in colony formation assays (Fig. 6A–E). These findings suggested the involvement of BNIP3-mediated mitophagy in CDDP resistance: BNIP3 silencing, per se, was sufficient to revert the mitophagic flux specifically induced by CDDP in resistant cells (Fig. 5) and to chemo-sensitize resistant cells (Fig. 6).

Second, we used PIK-III and SAR405, two inhibitors of the vacuolar sorting protein 34 (Vps34, a lipid kinase controlling autophagosomes formation): previously shown to increase sensitivity to chemotherapy [40–42]. After confirming that both inhibitors blocked autophagy (Fig. S6A–C) and validating their IC20 (Fig. S6B–D), they were used in combination with CDDP. We verified their potential to modulate sensitivity of resistant cells to CDDP cytotoxicity through colony formation assay. Our data showed that these inhibitors increased CDDP effect both in sensitive and in resistant lines, (Fig. 6F–H). Indeed, the Surviving Factor (number of colonies that arise after treatment) (Fig. 6G–I) showed a significant reduction of clonogenic survival in ovarian cancer resistant cells C13 with CDDP 1,5 µM and in osteosarcoma cells U2OS-PT with CDDP 3,1 µM in combination both with PIK-III and SAR405.

## Discussion

We have demonstrated that BNIP3-mediated mitochondrial clearance is a key mechanism underlying resistance to cisplatin (CDDP). Despite CDDP is a chemotherapeutic drug used for the treatment of a wide array of cancers, its clinical efficacy is limited by the onset of resistance phenotypes, whose underlying mechanisms are yet unclear [43, 44].

The pharmacological approaches for cancer treatments rely on the commonsense hypothesis that enhanced cell growth and replication should be counterbalanced by higher rates of programmed cell death. Thus, apoptosis is one of the most targeted pathways by the current therapies. Mitochondrial dynamics contribute differently to diverse types of cancer: it is still controversial whether mitochondrial fission or fusion promote cancer progression or apoptosis. To address this open question, we measured the expression of key players of cell cycle and cell death in our in vitro models of CDDP resistance. We found that both ovarian and osteosarcoma CDDP resistant cells are characterized by lower levels of p21-p53, suggesting dysregulation of the cell cycle, but not of the pro-apoptotic proteins BAX and BID nor of the ER stress markers ATF4 and GRP78.

As confirmed in literature, mitochondria fragmentation is a pre-requisite of mitophagy, favoring the engulfment of dysfunctional organelles by autophagosomes [45, 46]. Accordingly, we observed that in basal conditions ovarian and osteosarcoma CDDP resistant cells are characterized by a fragmented mitochondrial phenotype, associated with increased expression of the pro-fission proteins Drp-1, h-FIS1, MFFs and downregulation of the mitochondrial fusion mediators OPA1 and Mfn1. We found an opposite trend for the homologue of Mfn1: Mfn2. This could be ascribed to the additional, fusion-independent, role of Mfn2 in tethering the ER to the mitochondria [30]. Juxtaposition between these organelles is involved in many cell processes including mitochondrial fission and formation of autophagosomes [9, 16]. In our CDDP resistant models, both the proximity and the number of these sites of contact are increased, thereby providing an expanded platform for mitochondrial fission and autophagosome biogenesis [47]. A well-recognized therapeutical approach is sensitizing the cancer cells to chemotherapy through inhibition of early autophagic steps [40, 48], since autophagy and mitophagy are known to promote tumor progression [49] and to have cytoprotective effects from different anticancer agents [50]. The interplay between resistance to platinum drugs and autophagy is still unclear. It is still debated how positive or negative feedbacks between autophagy/mitophagy programs and pro-survival/pro-death apoptosis signaling are regulated. Thus, elucidating whether enhanced mitophagy can limit the mitochondrial ability to amplify chemotherapy-induced apoptosis can help to overcome resistance in cancer treatment. Interestingly, we uncovered for the first time that the key adaptor molecule for the mitophagic machinery BNIP3 has higher expression levels in CDDP resistant cells, suggesting its potential role in chemotherapy effectiveness response. Besides, applying a CDDP combinatory treatment with two inhibitors of the key autophagy regulator Vps34 reverted CDDP resistance.

Different in vivo studies reported contradicting roles for mitophagy receptors and signaling regulators in cancer, as the case of Chourasia et al. [27]. In this work, the authors showed that BNIP3 deletion in murine breast cancer promoted malignancy caused by accumulation of dysfunctional mitochondria and by angiogenesis induced by the increased oxidative stress. Nevertheless, many other cancer types presented a correlation between their aggressive phenotype and high BNIP3 expression. This can be due to simultaneous participation of mitophagic receptors in other mitophagy-independent signaling, e.i. their regulatory crosstalk with apoptosis, as the case of BNIP3.

Consistent with observations in the preclinical data, we have analyzed the association of BNIP3 expression with patient survival in two groups of ovarian cancer patients: we found that high BNIP3 levels in tumors partially resistant to platinum-based therapy were associated with significantly lower survival, in terms of progression-free survival. These data suggest that it could be exploited as a predictive marker for classified responsive patients to platinum-based chemotherapy. Correspondingly, the transcription factor HIF-1α, that controls BNIP3 mitophagic activity, was increased. Indeed, besides being able to inhibit c-Myc, a transcription factor that promotes mitochondrial biogenesis, HIF-1α is able to reduce mitochondrial mass and oxygen consumption by promoting mitophagy [37]. Altogether, these findings highlight that enhanced mitochondrial autophagy is a hallmark of CDDP resistant cells. Despite some evidence implied autophagy in protecting cancer cells from the lethal effects of CDDP-induced DNA damage [51, 52], and others linked BNIP3 proapoptotic role to cisplatin-induced cell death [53], the ultimate connection between these two processes has never been explored. Here we extended the role of BNIP3 in the context of CDDP resistance, highlighting that BNIP3-dependent mitochondrial autophagy is exploited by resistant cells to escape CDDP cytotoxicity. Consistently, we demonstrated that BNIP3 ablation re-sensitizes resistant clones to CDDP, by blunting mitochondrial autophagy. Our data indicate that BNIP3 itself, and more in general mitophagy, can be exploited as targets for the development of new therapeutical strategies to counteract CDDP resistance and its clinical relevance.

## Experimental procedures

### Chemotherapy agents and inhibitors

CDDP for in vitro experiments was purchased from *Teva*. PIK-III (#S7683) and SAR405 (#S7682) were purchased from *Selleckchem*. Bafilomycin A1 was purchased from Sigma-Aldrich (B1793–2UG).

### Cell lines

#### Human cancer cell lines (ovarian carcinoma; osteosarcoma)

Ovarian carcinoma (2008) sensitive and (C13) cisplatin-resistant cells were grown in Roswell Park Memorial Institute medium (RPMI 1640) (Lonza) and osteosarcoma U2OS and U2OS-PT cells were grown in McCoy’s 5 A (Lonza) medium, both media were supplemented with 10% fetal bovine serum (FBS), 4 mM glutamine, 100 U/ml penicillin and 100 µg/ml streptomycin, in humidified condition at 5% CO_2_ and 37 °C. For starvation treatment, cells were washed four times and then incubated in Hanks balanced salt solution (HBSS, *Lonza*) supplemented with 10 mM HEPES at pH 7.4, at 37 °C for the indicated time.

### Cell viability by trypan blue exclusion assay

In total, 1 × 10^5^ cells (2008, C13) or 2 × 10^5^ cells (U2OS, U2OS-Pt) were plated on 12 wells plates, and, following overnight incubation, exposed to different treatments, according to experimental protocols. At the end of incubation, the cells were washed, detached with 0.25% trypsin-0.2% EDTA and suspended in trypan blue (*Sigma-Aldrich*, St Louis, MO, USA), at 1:1 ratio in medium solution. Cell number was counted using a chamber Burker hemocytometer.

### Protein extraction and immunoblot assay

In total, 1.5 × 10^6^ cells (2008, C13) or 1 × 10^6^ cells (U2OS, U2OS-PT) were plated in 100 mm cell culture dish and allowed to attach overnight. After 48 h, cells were lysed with ice-cold lysis buffer [TRIS 25 mM pH 7,4; NaCl 150 mM; IGEPAL 1%; sodium deoxycholate 1%; SDS 0,1%; EDTA 1 mM] supplemented with the protease inhibitor cocktails (*Roche Molecular Biochemicals*, Mannheim, Germany). Cell lysates were then centrifuged at 14,000 g for 15 min at 4 °C and the supernatant protein content was determined by Lowry procedure (*Bio-rad DC* Protein Assay) using bovine serum albumin as standard. Equal amounts of protein (30 μg) were loaded on a polyacrylamide gel and electrophoretically separated in running buffer. After electrophoresis, the proteins were blotted onto an Hybond-P PVDF membrane (*Amersham Biosciences*, Buckinghamshire, UK). After blocking, the membrane was exposed to the elected primary antibodies: Mfn2 (1:1000; Rabbit, *Abnova*, H00009927-M03), Mfn1 (1:1000; Mouse, *Abcam*, ab60939–100), OPA1 (1:1000; Rabbit, *BD-Biosciences*, 612607), LC3B (1:1000; Rabbit, *Cell Signaling*, 2775), p-DRP1 (1:1000; Rabbit, *Cell Signaling*, 4867 S), total DRP1 (1:1000, Mouse, *BD Transduction*, 611738) BNIP3 (1:1000, Rabbit, *Abcam*, ab109362) and p62 (1:1000, Rabbit, MLB, P0067), MFF (1:1000, Rabbit, *ProteinTech*, 17090–1-AP), p53 (1:1000, Rabbit, *Cell Signaling*, 9287 S), p21 Waf1/Cip1 (1:1000, Rabbit, *Cell Signaling*, 2947), BAX (1:1000, Mouse, *Abcam*, ab5714), BID (1:1000, Mouse, *R&S System*, MAB860), HIF-1α (1:1000, Rabbit, *Novus*, NB-100–134), c-MYC (1:1000, Mouse, *Sigma*, MA1–980), VDAC1 (1:1000, Mouse, *Abcam*, ab15895), OXPHOS (1:1000, Mouse, *Abcam*, ab110413), GRP75 (1:1000, Rabbit, *Santa Cruz*, sc13967), CYD (1:1000, Mouse, *Abcam*, ab110324), COX IV (1:1000, Mouse, *Abcam*, ab14705), ATF4 (1:1000, Mouse, *Abnova*, H00000468-M01), GRP78 (1:1000, Rabbit, *BD-Biosciences*, 610978), PINK1 (1:1000, Rabbit, *Novus*, BC100–494), PARKIN (1:1000, Mouse, *Santa Cruz*, sc-32282), phospho-PARKIN (1:1000, Rabbit, *Cell Signaling*, cst-36728), AMBRA1 (1:1000; Mouse, *Santa Cruz*, sc398204) overnight at 4 °C. After washing, the membrane was incubated with HRP-conjugated anti-rabbit secondary antibody (1:3500; *PerkinElmer*, MA, USA) and HRP-conjugated anti-mouse secondary antibody (1:10000, *Dako*). The signal was visualized with enhanced chemiluminescent kit (*Amersham Biosciences*) according to the manufacturer’s instructions and analyzed by Molecular Imager VersaDoc MP 4000 (*Bio-rad*). The integrated intensity was normalized to antibodies: Tom20 (1:2000; Rabbit, *Santa Cruz*, FL-145), beta-actin (1:5000; Mouse, *Abcam, ab8226*), calnexin (1:2000; Rabbit, *ENZO*, ADI-SPA-860-F).

### Quantitative Real Time PCR analysis

Cells were grown as indicated and total mRNA was extracted as per manufacturer’s instructions using kit Direct-zol™ RNA MiniPrep (*Zymo research*) and measured with a NanoDrop 2000 (*Thermo Fischer Scientific Inc*.). The relative expression of each gene was determined by quantitative real-time PCR (Eco™Illumina, Real-Time PCR system, San Diego, CA, USA) using One Step SYBR PrimeScript RT-PCR Kit (*Takara Bio*, Inc., Otsu, Shiga, Japan) and the primers designed as follow:

BNIP3: F: 5′-GAATTTCTGAAAGTTTTCCTTCCA-3′,

R: 5′-TTGTCAGACGCCTTCCAATA-3′;

H-FIS1: F: 5′-CTTGCTGTGTCCAAGTCCAA-3′,

R: 5′-CCACAGCCCCGTTTTATTTA-3′;

DRP1: F: 5′-CAGTGTGCCAAAGGCAGTAA-3′,

R: 5′-GATGAGTCTCCCGGATTTCA-3′

MFN2: F: 5′- GACCCCGTTACCACAGAAGA-3′,

R: 5′- GCAGAACTTTGTCCCAGAGC-3′

OPA1: F: 5′- CCACAGATTTCTCCCAAGGA-3′,

R: 5′- CCCATGAAAGAAGCCACATT-3′

PINK1: F: 5′- GAA GCC ACC ATG CCT ACA TT-3′,

R: 5′- CTC CTG GCT CAT TGT GTT CA-3′

PARKIN: F: 5′- CAG TTT GTT CAC GAC CCT CA-3′,

R: 5′- TTC GCA GGT GAC TTT CCT CT-3′

PARL: F: 5′- GGG TAA AGT TGC CAC AGG AA-3′,

R: 5′- ATG GCG ATA ATG GCT TTC AG-3′

Linearity and efficiency of PCR amplifications were assessed using standard curves generated by serial dilution of complementary DNA; melt-curve analysis was used to confirm the specificity of amplification and absence of primer dimers. All genes were normalized to β-ACTIN:F:5′-CCAACCGCGAGAAGATGA-3′,R: 5′-CCAGAGGCGTACAGGGATAG-3′ for 2008-C13 cell lines and CALNEXIN: F: 5′-GAAGGGAAGTGGTTGCTGTG-3′; R:5′-GATGAAGGAGGAGCAGTGGT-3′ for U2OS-U2OS-PT. Expression levels of the indicated genes were calculated by the ΔΔCt method using Eco™ Software v4.0.7.0.

### Imaging

#### Mitochondrial network

Cells were seeded on 13 mm round glass coverslips at approximately 50% confluence in a 12 well/plate and incubated overnight. After 48 h, cells were fixed with 4% formaldehyde (*SIGMA*, St Louis, USA) for 20 min. Then, cells were exposed to Triton 0.1% for 5 min and then to blocking solution in PBS with FBS 7% and NaN_3_ 0.02%. Cells were stained with TOM20 antibody (1:300; Santa Cruz) and with anti-rabbit secondary antibody conjugated with Alexa fluor 488 (1:500; *Invitrogen*). Epifluorescence images acquisition was performed in the Zeiss Confocal Microscope (LSM700 microscope). Images were acquired with 63X objective for osteosarcoma cells and 100X for ovarian cancer cells and 15 planes were acquired in z-stack with a distance of 0.4 μm between each plane for each cell. The following filter was utilized: 488 (Ex. 490–510 / Em. 520–560). After acquisition, images were analyzed using ImageJ analysis software. For analysis of mitochondrial morphology, mitochondria segmentation was performed using the ImageJ Squassh [54] plugin, and size and morphology features were performed using Fiji.

#### FRET assay and mitochondria–ER contact sites

Cells were seeded at 2 x 10^3^ cells/well in a 384 well/plate (μclear-plate black, Greiner Bio One) and incubated overnight. The day after, after incubation with 8 μg/ml polybrene for 30 min, cells were transduced with Adenovirus Ad(RGD)-CMV-FEMP-YFP (*Vector Biolabs*) kindly provided by Dorn G.W.2nd (Italian Ministry of Health authorization number: PD/IC/Op2/19/001). The modified FEMP construct was produced as previously described [31]. Imaging was performed 48 h after transfection. After imaging in resting conditions, the medium was removed from the 384 wells plate and fresh medium containing Rapamycin at a final concentration of 100 nM was added and let incubate for 15 min at RT. Post incubation, the Rapamycin-containing medium was removed, and cells were fixed by addition of 1% Formaldehyde (*SIGMA*) incubating for 15 min at RT. Subsequently, formaldehyde is removed and 90 μl of PBS are added in each well. Image acquisition was performed in Operetta® High Content Imaging System (*Perkin Elmer*) and the settings were established with the Harmony 3.5 software. Images were acquired with 20X objective. The following filters were utilized: CFP (Ex.410–430/ Em.460–500), YFP (Ex.490–510/Em.520–560) and YFP-FRET (Ex.410–430/Em.520–560). After acquisition, images were analysed using Perkin Elmer Harmony 3.5 image analysis software. The YFP channel was chosen to mark the region of interest (ROI) and around each ROI, a second boundary was drawn to measure the background intensity. FRET basal and FRET max were calculated as: (FYFP FRETcell-FYFPFRETbg)/ (FCFPcell-FCFPbg); FRET Ratio was calculated as (FRETmax–FRETbasal)/FRETbasal. High Content Imaging was performed at the HiTS@Unipd facility, Dept. of Biology, University of Padua.

#### Colony formation assay

Cells were plated at a density of 50 cells/well in 96-well plates, eventually transfected for BNIP3 silencing and treated with compounds (PIK-III, SAR405 and CDDP in combination or alone) the following day. Then, colonies were stained with Cell Traker Red CMPTX dye (*Life Technologies*, #C34552) and with 25 mg/mL Hoechst 33342 (*SIGMA*, #B2261) followed by washing steps. Image acquisition was performed in Operetta® High Content Imaging System (*Perkin Elmer*) using a 10X or 20X objective in widefield fluorescence mode in 2 fluorescent channels (Ex.360–340/Em.410–480, for Hoechst 3334; and Ex. 557/Em.602 for Cell Traker Red CMPTX) and further analysed using Perkin Elmer Harmony 3.5 image analysis software. “Find Nuclei Building Block” algorithm was applied to identify Hoechst-33342-labeled nuclei. “Find Spots Building Block” was applied to identify cells for measurement of colony formation. “Calculate Intensity Properties Building blocks” was used to determine cell number and colonies. The PE factor (ratio of the number of colonies to the number of cells seeded) and the SF factor (number of colonies that arise after treatment of cells) were calculated as previously described [55].

#### mito-Keima imaging and mitophagy measurement

Cells were plated at a density of 1 x 10^3^ cells/well in 384-well plates, transfected for 48 h with a plasmid encoding for mito-Keima; we upscaled to high throughput settings the previously described mitophagy analysis (e.g. mitophagy index; [56]). The cells were treated with CDDP or FCCP (as a positive control). Mitophagic Indexes were calculated based on the fluorescence of mitochondria-targeted mKeima plasmid (mt-Keima) [57] (MBL International); courtesy of V. Romanello and M. Sandri as in [56]. The Index is defined as the total number of red pixels divided by the total number of all pixels corrected by the background. Fluorescence was imaged in two channels via two excitations (460–490, 520–550 nm excitations) and using a 640- to 680-nm emission range.

### Transmission Electron Microscopy and mitochondrial morphology

#### Sample preparation

24-multiwells plates were seeded with a constant number of cells and, following overnight incubation, cells were fixed with 2.5% glutaraldehyde in sodium cacodylate 0.1 M pH 7.4 for 1 hour at 4 °C, and then postfixed with 1% osmium tetroxide and 1.5% potassium ferrocyanide in 0.1 M sodium cacodylate pH 7.4 for 1 hour at 4 °C. Samples were dehydrated through a graded series of ethanol, infiltrated and then embedded in epoxy embedding medium (*Fluka*). After being stained with uranyl acetate and lead citrate, the sections were observed under a Tecnai-12 electron microscope (Philips-FEI) transmission electron microscope operating at 100 kV. Images were collected by a F114 (TVIPS) CCD camera. The TEM images and experiment are performed from the University of Padua electron microscopy facility.

### Mitochondrial Morphometry analysis and ERMICC

Mitochondrial parameters were measured using ImageJ (National Institutes of Health) by two different operators blinded to the identity of the sample. Mitochondrial perimeter and mito-ER contact sites were quantified with Image J Freehand line selection tool. Sample size is indicated in the figure legends. As reported in Naon et al 2016 [31], we used the ER–mitochondria contact coefficient (ERMICC) to measure the extent of physical interaction between mitochondria and ER. This index considers not only the distance between the ER and mitochondria but also the length of the surfaces juxtaposition and the perimeter of the mitochondria involved in the interaction. For calculations of mitochondria–ER distance, n > 3 mitochondria per image in 60 images per condition were considered, and a minimum distance of ER located in a 90–30 nm radius from the considered mitochondria was computed.

### BNIP3 silencing and cell death assay

Sensitive and resistant cells were plated in 12 well/plate at a density of 2.5 x 10^4^ cells/well (2008-C13) or 1 x 10^5^ cells/well (U2OS-U2OS-PT) in a final volume of 1 mL, upon harvesting with Trypsin 0.25% EDTA. Transfection complex was prepared in FBS free-Medium with esiRNA BNIP3 (Mission esiRNA, EHU112661–50UG, *SIGMA*) and of DharmaFECT transfection reagent (T-2004–03, *Dharmacon-Ge Healthcare*), according to the manufacturer instructions. The transfection mixture was dispensed at a concentration of 100 nM/well for 48 h. Then, cells were treated with cisplatin 1–5–10–25–50 μM for 24 h. Cell death was measured with Trypan Blue exclusion Assay as described above.

### Clinicopathological, immunoblot and immunohistochemistry analyses of patient’s samples

Informed consent was obtained from each patient with histologically confirmed epithelial ovarian cancer to be used for research. The study was approved by the Institutional Review Board of the CRO Aviano National Cancer Institute, Italy. Freshly frozen tumour samples were collected from patients with primary epithelial ovarian cancer who underwent surgical resection, before any chemotherapeutic treatment and at disease recurrence, at CRO Aviano. Samples for immunoblot were used by preparing lysates in RIPA buffer (see section of Protein extraction) and by performing homogenization with Tissue Lyser Machine (*Leica CM1850*). For the second group of patient’s samples, informed consent was taken from Istituto Nazionale Tumori and the study was approved by the Independent Ethics Committee of Istituto Nazionale Tumori-IRCCS. G. Pascale, Italy. The ovarian cancer tissues were analyzed in two patients: C.P (platinum-sensitive ovarian cancer) and B.A (platinum-resistant patient). Immunohistochemistry was performed on formalin-fixed, paraffin-embedded sections of ovarian tumor biopsies. Paraffin slides were then deparaffinized in xylene and rehydrated through graded alcohols. Antigen retrieval was performed with slides heated in 0.0.1 M citrate buffer (pH 8.0.) in a bath for 10 min a 110 °C. The slides were rinsed with TBS and the endogenous peroxidase was inactivated with 3% hydrogen peroxide. After protein block (BSA 5% in PBS 1×), the slides were incubated with primary antibody to human TIM23 (dilution 1:350, OTI2F5*, Invitrogen*), TOM20 (dilution 1:300, FL-145, *Santa-Cruz*), BNIP3 (dilution 1:300, ab109362*, Abcam*). Sections were incubated with mouse anti-rabbit or goat anti-mouse secondary IgG biotinylated secondary antibody for 30 min. Immunoreactivity was visualized by means of avidin–biotin–peroxidase complex kit reagents (*Novocastra, Newcastle, UK*) as the chromogenic substrate. Finally, sections were weakly counterstained with haematoxylin and mounted. The analysis of clinical samples was performed by experienced pathologists and scientist in a blinded manner based on the mitochondrial staining intensity (TIM23 and BNIP3 markers) (0, negative; 1, weak; 2, moderate; and 3, strong) and the proportion of stained tumor cells (0–100%).

### TCGA data retrieval and annotation

We retrieved processed RNA-seq data from the National Cancer Institute (NCI) Genomic Data Commons (GDC) repository. Specifically, we used the “TCGAbiolinks” R package [58] to download gene expression quantification (HTSeq - FPKM-UQ workflow) of all samples belonging to the TCGA-OV project. Clinical annotation was extracted from Table S1 of [36] and cross-referenced with RNA-seq measurements using the available patient barcodes.

### Statistical analyses

All data are expressed as mean ± SEM. A minimum of three independent experiments were performed with similar results. Standard ANOVA procedures followed by multiple pairwise comparison adjusted with Bonferroni corrections were performed for cell viability assays. Unpaired Student’s *t*-tests, two-sided, were used to analyze all the other results. Significance was considered at *p* < 0.05. Outliers were removed when not in the range of MEAN ± (1.5* S. D.).

### Supplementary information

Supplementary Experimental Procedures

Supplementary Figure Legends

Supplementary Figure 1

Supplementary Figure 2

Supplementary Figure 3

Supplementary Figure 4

Supplementary Figure 5

Supplementary Figure 6

Supplementary Figure 7

Reproducibility Checklist

## Data Availability

The datasets generated and/or analyzed during the current study are available from the corresponding author on reasonable request.
