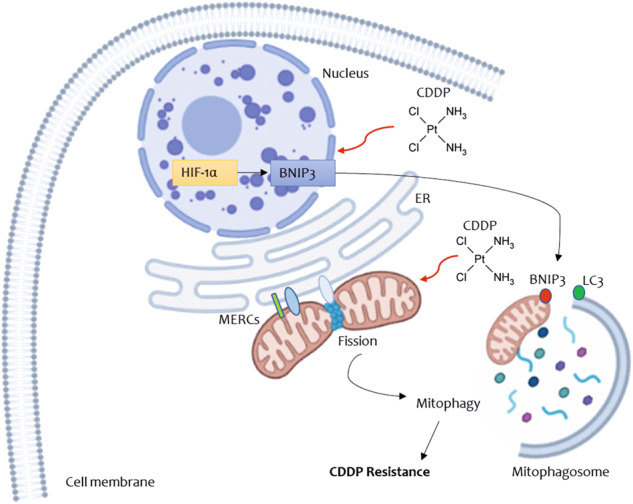# Correction: Cisplatin resistance can be curtailed by blunting Bnip3-mediated mitochondrial autophagy

**DOI:** 10.1038/s41419-022-04905-7

**Published:** 2022-05-09

**Authors:** Caterina Vianello, Veronica Cocetta, Daniela Catanzaro, Gerald W Dorn, Angelo De Milito, Flavio Rizzolio, Vincenzo Canzonieri, Erika Cecchin, Rossana Roncato, Giuseppe Toffoli, Vincenzo Quagliariello, Annabella Di Mauro, Simona Losito, Nicola Maurea, Cono Scaffa, Gabriele Sales, Luca Scorrano, Marta Giacomello, Monica Montopoli

**Affiliations:** 1 Department of Pharmaceutical and Pharmacological Sciences, University of Padova, Largo E. Meneghetti 2, 35131 Padova, Italy; 2 Department of Biology, University of Padova, Via Ugo Bassi 58B, 35131 Padova, Italy; 3 Center for Pharmacogenomics, Department of Internal Medicine, Washington University School of Medicine, 660 S. Euclid Ave., St. Louis, MO 63110 USA; 4 Sprint Bioscience, Huddinge, Sweden; 5 Department of Oncology-Pathology, Karolinska Institute, Stockholm, Sweden; 6 Department of Molecular Sciences and Nanosystems, Ca’ Foscari University of Venice, 30172 Venice, Italy; 7 Pathology Unit, Centro di Riferimento Oncologico di Aviano (C.R.O.) IRCCS, 33081 Aviano, Italy; 8 Department of Medical, Surgical and Health Sciences, University of Trieste, 34149 Trieste, Italy; 9 Experimental and Clinical Pharmacology Unit, Centro di Riferimento Oncologico (CRO), IRCCS, 33081 Aviano, Italy; 10 Division of Cardiology, Istituto Nazionale Tumori-IRCCS-Fondazione G. Pascale, Naples, Italy; 11 Pathology Unit, Istituto Nazionale Tumori-IRCCS-Fondazione G. Pascale, Naples, Italy; 12 Gynecologic Oncology, Istituto Nazionale Tumori-IRCCS-Fondazione G. Pascale, Naples, Italy; 13 Veneto Institute of Molecular Medicine, Via Orus 2, 35129 Padova, Italy; 14 Department of Biomedical Sciences, Via Ugo Bassi 58B, 35131 Padova, Italy

**Keywords:** Cancer therapeutic resistance, Mitophagy

Correction to: *Cell Death and Disease* 10.1038/s41419-022-04741-9, published online 22 April 2022

The original version of this article unfortunately contained a mistake. Due to a typesetting error the graphical abstract was omitted. We apologize for the error. The graphical abstract can be found below. The original article has been updated.