# Supplementary material for: Cisplatin resistance can be curtailed by blunting Bnip3-mediated mitochondrial autophagy
Source: Cell Death Dis. 2022 Apr 22;13(4):398. doi: 10.1038/s41419-022-04741-9 (PMC9033831; doi:10.1038/s41419-022-04741-9)
Supplement: Supplementary file 1 — Supplementary Experimental Procedures [file 41419_2022_4741_MOESM1_ESM.docx]

**SUPPLEMENTARY MATERIAL**

***Supplementary Experimental procedure***

***1. Flow cytometry***

2.5×10^5^ cells (2008, C13) or 2×10^5^ cells (U2OS, U2OS-Pt) were plated on 6 wells plates, and, following overnight incubation, exposed to different treatments, according to experimental protocols. At the end of incubation, the cells were washed, detached with 0.25% trypsin-0.2% EDTAand centrifuged for 5 min at 1200 rpm. Cells were resuspended with 20 nM Mitotracker Green (MTG; *Invitrogen*, #M7514) or nonyl acridine orange (NAO) (25 nM) or Rhodamine123 (10 µM) and incubated for 20 minutes. Fluorescence intensity was analyzed using an BD FACS Canto II flow cytometer (VIMM Flow cytometry Facility). 10000 cells of interest were analyzed. Mean fluorescence intensity [MFI] values were obtained using the BD FACS Diva Software.

***2. Immunofluorescence of patients’ tissues***

The ovarian cancer tissues were analyzed in two patients: C.P (platinum-sensitive ovarian cancer) and B.A (platinum-resistant patient). Immunofluorescence was performed on formalin-fixed sections of ovarian tumor biopsies. After their dewaxing and hydration, the prepared slides were incubated with antigen recovery in a pressure cooker (*Biocare*) for 10 minutes at 110 ° C. After blocking with BSA 5% in PBS 1×, the slides were incubated first with primary antibody to human TIM23 (dilution 1:350, OTI2F5, *Invitrogen*), TOM20 (dilution 1:700, FL-145, *Santa-Cruz*), BNIP3 (dilution 1:300, ab109362, *Abcam*), and then were detected using a cocktail of dylight 488 goat anti-mouse IgG and dylight 594 goat anti-rabbit IgG (dilution 1:250, *Abcam*). After washing three times, the nuclei were stained with DAPI at room temperature for 10 minutes and stored at 4 ° C. The slides were examined using a fluorescence microscope (*Olympus BX61*).
