# Supplementary material for: Cisplatin resistance can be curtailed by blunting Bnip3-mediated mitochondrial autophagy
Source: Cell Death Dis. 2022 Apr 22;13(4):398. doi: 10.1038/s41419-022-04741-9 (PMC9033831; doi:10.1038/s41419-022-04741-9)
Supplement: Supplementary file 2 — Supplementary Figure Legends [file 41419_2022_4741_MOESM2_ESM.docx]

**SUPPLEMENTARY FIGURES**

***Supplementary figure 1: Characterization of the mitochondrial phenotype in CDDP resistant human cancer cells***

**(C)** IC50 values for each cell line, obtained from concentration-response curves after 24h of cisplatin treatment **(A)** and **(B)**. Data are the mean ± SEM of 5 different experiments. *p<0.05; **p<0.01; ***p<0.001; calculated by a two-tailed unpaired t-test comparing resistant *vs* sensitive cells. **(D)** Mitochondrial mass and membrane potential measured by flow cytometry and expressed as ratio of Mean Fluorescent Intensity [MFI] of NAO (25 nM) and Rh123 (10 µM) between U20S PT resistant cells and their sensitive counterpart. Each bar represents the mean ± SEM of 3-4 independent experiments. A similar analysis was performed in 2008 and C13 cells, and it is described in (^16^) and (^59^). **(E)** Effect of galactose (5mM) and **(F)** rotenone (0.1-10 µM) on U2OS/U2OS-PT cells incubated for 24 hours in complete DMEM or in glucose free/galactose medium. Data are expressed as % of cell number as compared to the control. Data are the mean±SEM of 3 different experiments; +++*p<*0.001, calculated by a two-tailed unpaired t-test comparing galactose treated cells *vs* not treated cells. A similar analysis was performed in 2008 and C13 cells, and it is described in (^16^) and (^59^). **(G-I-K-M)** Mitochondrial proteins (TOM20, CYD, VDAC1, GRP75, OXPHOS complexes) expression. Optical density (O.D., arbitrary unit) was normalized to β-actin for 2008-C13 **(H)** or calnexin for U2OS-U2OS-PT **(J)**. **(L)** and **(N)** OXPHOS mitochondrial complexes expression was normalized to TOM20**.** Data are expressed as ratio between resistant and sensitive cell lines. Data are the mean±SEM of 4 independent experiments. **p<*0.05, ***p<*0.01 calculated by a two-tailed unpaired t-test comparing resistant *vs* sensitive cells.

***Supplementary figure 2: ER stress and apoptosis markers are not significantly changed in resistant clones as compared to sensitive cells***

**(A)** and **(C)** Expression of ATF4 and GRP78 in basal condition and after CDDP treatment (1µM-10µM). The optical density (O.D. arbitrary unit) was normalized respectively to β-actin for 2008-C13 **(B)** and to calnexin for U2OS-U2OS-PT **(D)**. The data are expressed as the ratio of treated cells *vs* untreated samples. Data are the mean±SEM of 3 different experiments. **(E)** and **(G)** Expression of p53, p21, BAX, BID. The optical density (O.D.) was normalized respectively to β-actin for 2008-C13 **(F)** and to calnexin for U2OS-U2OS-PT **(H)**. The data are expressed as ratio of resistant *vs* sensitive cells. Data are the mean±SEM of 3 different experiments. ****p<*0.001, calculated by a two-tailed unpaired t-test comparing resistant *vs* sensitive cells.

***Supplementary figure 3: Mitochondrial mass does not increase in resistant cells during starvation and upon CDDP treatment***

**(A)** and **(C)** Expression of TOM20, Cyclophilin D and COX IV after 8h starvation in HBSS, the optical density was normalized respectively to β-actin for 2008-C13 and to calnexin for U2OS-U2OS-PT. In **(B)** and **(D)** the data are expressed as the ratio of treated cells to untreated cells. Data are the mean±SEM of 3-4 different experiments. **p<*0.05, calculated by a two-tailed unpaired t-test comparing resistant *vs* sensitive cells. **(E)** and **(G)** Effect of 24h of CDDP (1µM) on mitochondrial mass of 2008-C13 and U2OS and U2OS-PT, by the expression of mitochondrial proteins TOM20, Cyclophilin D and COMPLEX IV. The optical density was normalized respectively to β-actin for 2008-C13 and to calnexin for U2OS-U2OS-PT. In **(F)** and **(H)** the data are expressed as the ratio of treated cells to untreated cells. Data are the mean±SEM of 3-4 different experiments. **p<*0.05, calculated by a two-tailed unpaired t-test comparing resistant *vs* sensitive cells. **(I)** Mitochondrial mass measured by flow cytometry and expressed as [MFI] % of Mitotracker Green (MTG) (20 nM) on control. Cells were exposed to HBSS for 8h and CDDP 1µM for 24h. Each bar represents the mean ± SEM of 4 independent experiments. *p<0.05, calculated by a two-tailed unpaired t-test comparing treated vs untreated cells.

***Supplementary figure 4: BNIP3 is expressed in ovarian cancer patients resistant to CDDP***

**(A)** Representative images showing BNIP3, TIM23 protein expression and DAPI determined by immunofluorescence staining in ovarian cancer patient’s tissues. (B) Representative images showing BNIP3, TIM23 protein expression determined by immunofluorescence staining in ovarian cancer patient’s tissues. (C) Representative images showing BNIP3, TOM20 protein expression and DAPI determined by immunofluorescence staining in ovarian cancer patient’s tissues. **(D)** BNIP3 protein expression analyzed by a bioinformatic analysis using the RNA-seq data generated by the TCGA consortium (as in Figure **3M**). The graph represented the analysis of PFS in association with BNIP3 expression levels in the three groups of patients (too early, resistant and sensitive as defined in ^36^). Resistant patients presented high BNIP3 gene expression associate with low progression-free survival (PFS) compared to sensitive patients.

***Supplementary figure 5: BNIP3, but not other mitophagic markers, contributes to CDDP resistance in ovarian and osteosarcoma cells***

(**A**) AMBRA1, PINK1, phospho-PARKIN (p-PARKIN) and PARKIN total (PARKIN TOT) protein expression. (**B**) Optical density (O.D.) was normalized to TOM20 for AMBRA, PINK1 and p-PARKIN; for total PARKIN, to β-actin for 2008-C13 and to calnexin for U2OS-U2OS-PT. Data are the mean±SEM of 3 different experiments; *p<0.05, **p<0.01, ***p<0.001, calculated by a two-tailed unpaired t-test comparing resistant *vs* sensitive cells. (**C**) mRNA expression of PINK1, PARKIN, protease PARL (presenilin associated rhomboid-like protein) genes normalized to β-actin (2008 and C13) and calnexin (U2OS and U2OS-PT). The data are expressed as the ratio of resistant to sensitive cells set to 1. *p<0.05, calculated by a two-tailed unpaired t-test comparing resistant *vs* sensitive cells. **(D)** Quantification of Mitophagic Index upon overnight treatment with FCCP (10µM) of 2008-C13 cells and U2OS-U2OS-PT cells. Data are the mean±SEM of 5 different experiments. *p<0.05; **p<0.01; calculated by a two-tailed unpaired t-test comparing treated *vs* untreated cells. **(E)** and **(F)** BNIP3 silencing reduces the effect of CDDP and BAF-A1 treatments in resistant cells. Effect of 24h of CDDP (1µM) and bafilomycin A1 (100nM) on BNIP3 and P62 expression of 2008-C13 and U2OS-U2OS-PT transfected with a control NTC esiRNA (scramble esiRNA) or esiRNA targeting BNIP3 (esiBNIP3). BNIP3 protein expression normalized to TOM20, β-ACTIN (2008-C13) and calnexin (U2OS-U2OS-PT). The respective quantification is reported in **(G)**. The data are expressed as the ratio of treated cells with respect to untreated scramble cells. Data are the mean±SEM of 3-4 different experiments. *p<0.05 calculated by a two-tailed unpaired t-test comparing treated *vs* untreated cells.

***Supplementary figure 6: The inhibitors PIK-III and SAR-405 block autophagy in ovarian and osteosarcoma cells***

Autophagic inhibition was measured by assessing the levels of p62, LC3 BI, LC3 BII after 24h of PIK-III and SAR-405 (1-10 µM) treatment. 200nM of BafA1 was added for the last 3 hours of treatment. The optical density was normalized on actin (2008-C13) **(A)** or calnexin (U2OS-U2OS-PT) **(C)**. IC20 of PIK-III and SAR-405 (1-10 µM) values and curves of 2008-C13 in **(B)** and U2OS-U2OS-PT in **(D)**. The data are expressed in percentage of treated cells respect to untreated cells. Data are the mean±SEM of 2 different experiments.

***Supplementary figure 7: Uncropped original western blots***

**Reference**

59. Montopoli M., Bellanda M., Lonardoni F., Ragazzi E., Dorigo P., Froldi G., et al. Metabolic reprogramming in ovarian cancer cells resistant to cisplatin. *Bentham Science Publishers* 2011; **11**(2): 226–235.
