# Supplementary material for: Cisplatin resistance can be curtailed by blunting Bnip3-mediated mitochondrial autophagy
Source: Cell Death Dis. 2022 Apr 22;13(4):398. doi: 10.1038/s41419-022-04741-9 (PMC9033831; doi:10.1038/s41419-022-04741-9)

Original uncropped western blots of Figure 1

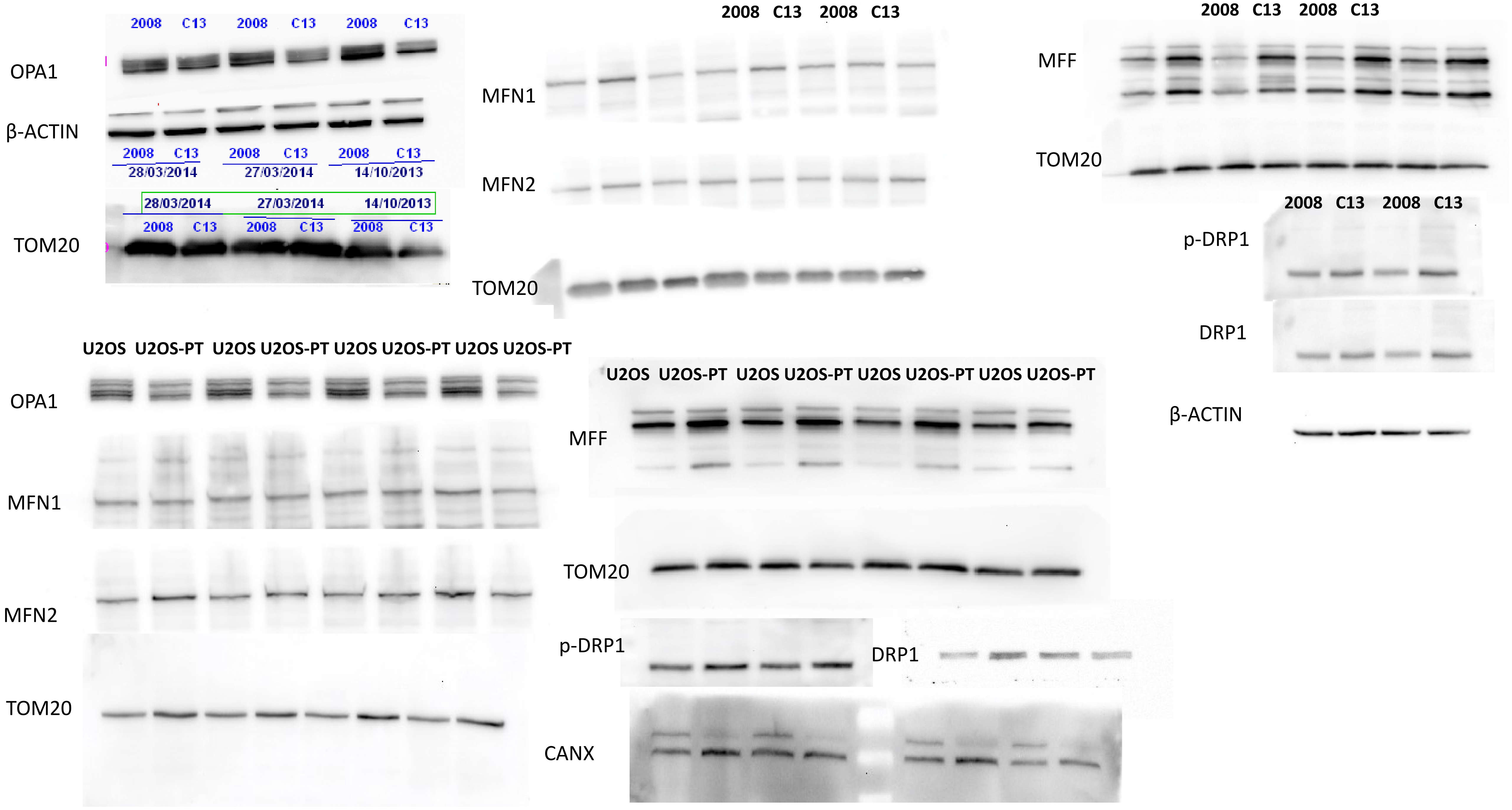

Original uncropped western blots of Figure 3A

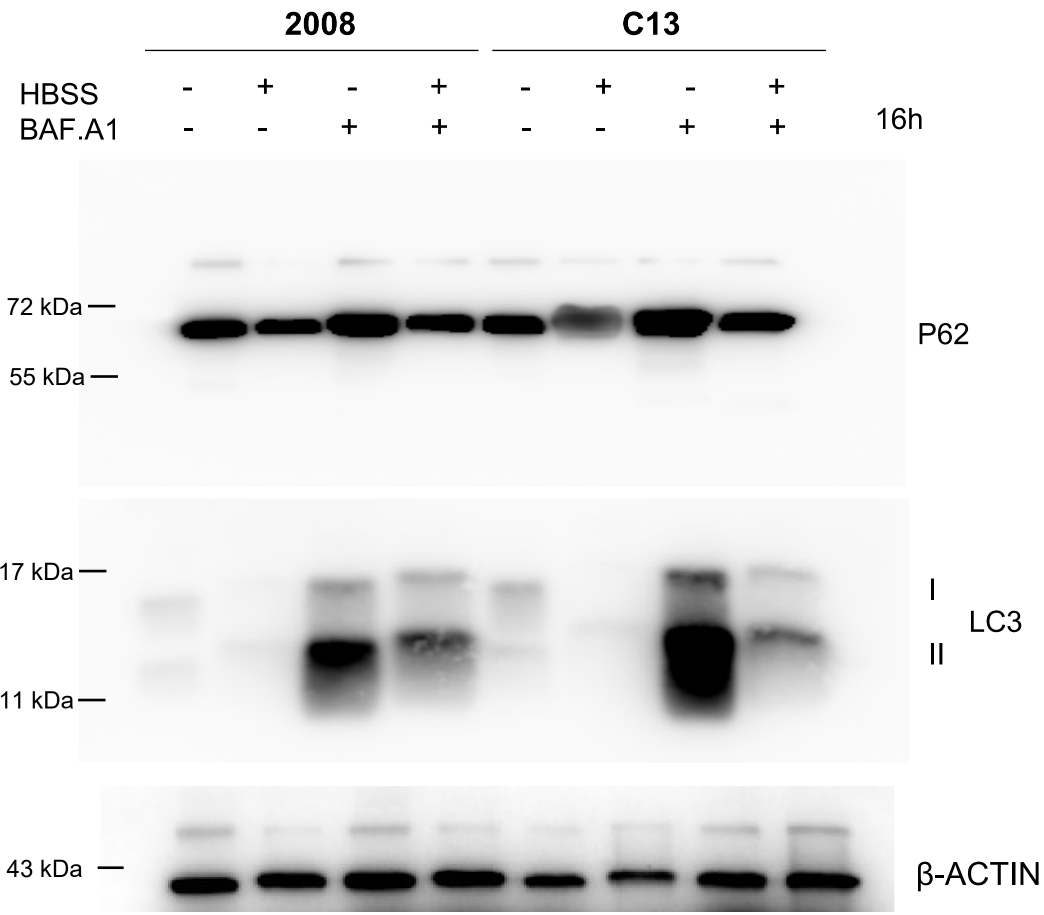

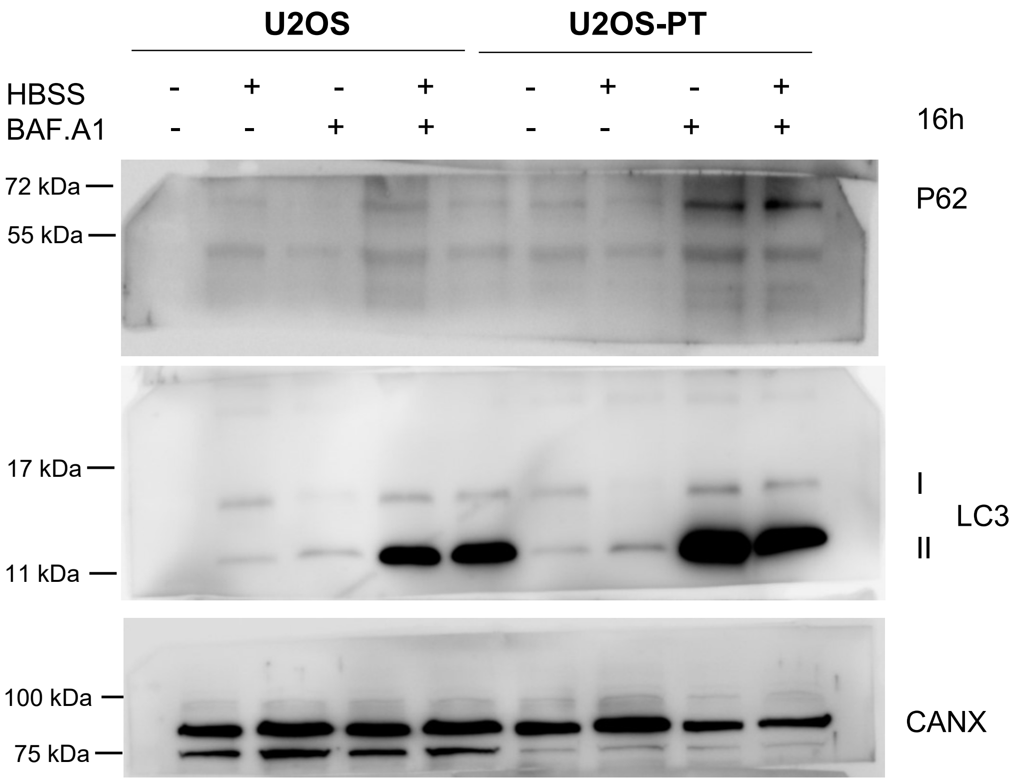

Original uncropped western blots of Figure 3B

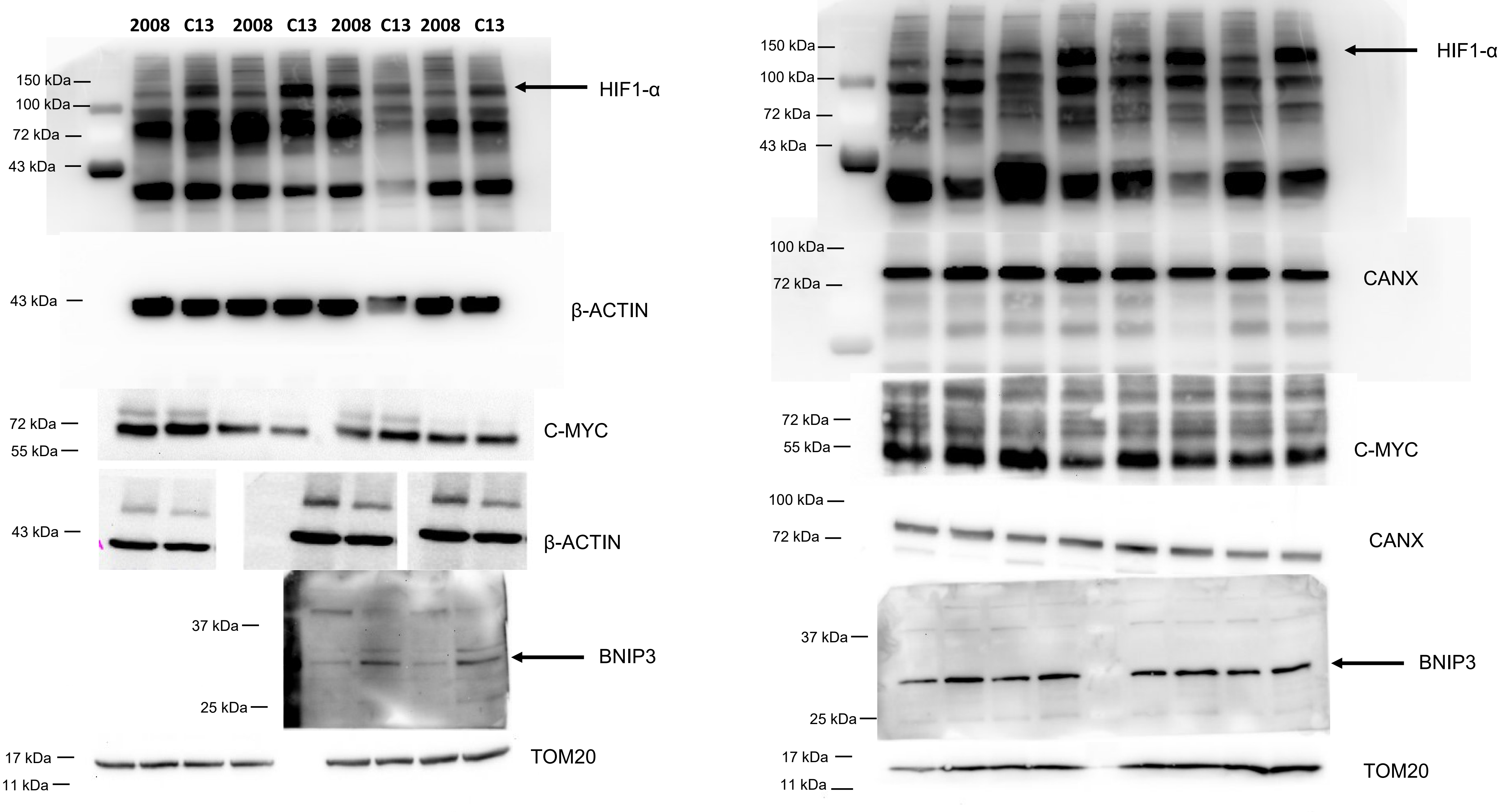

Original uncropped western blots of Figure 3C

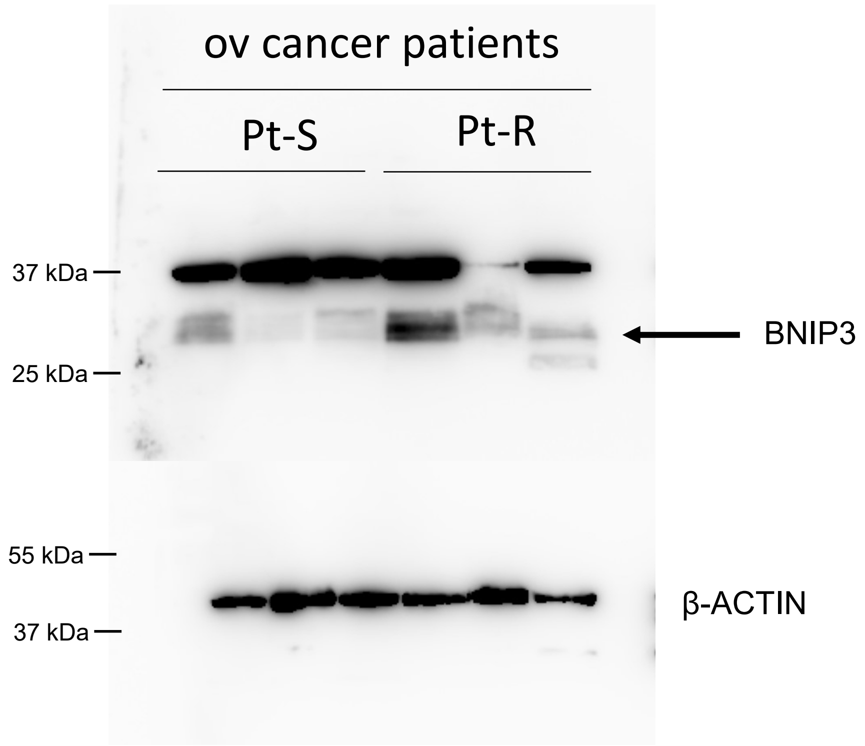

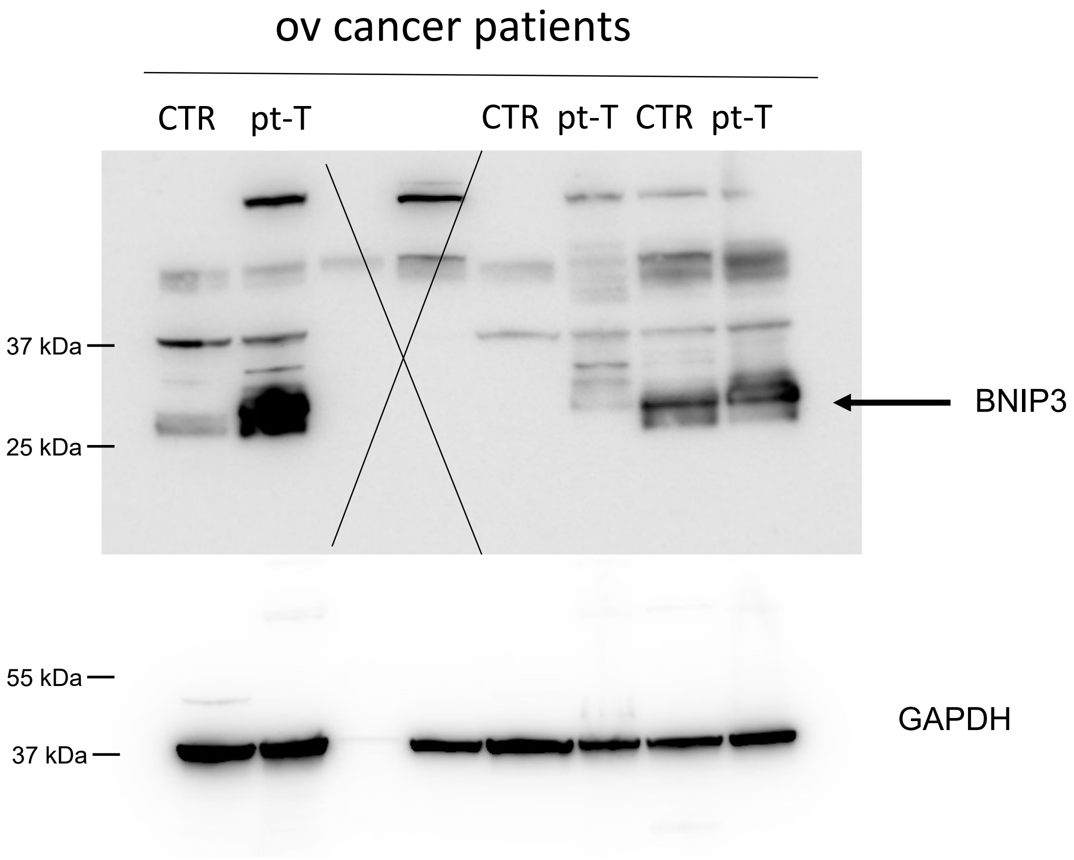

Original uncropped western blots of Figure 4A

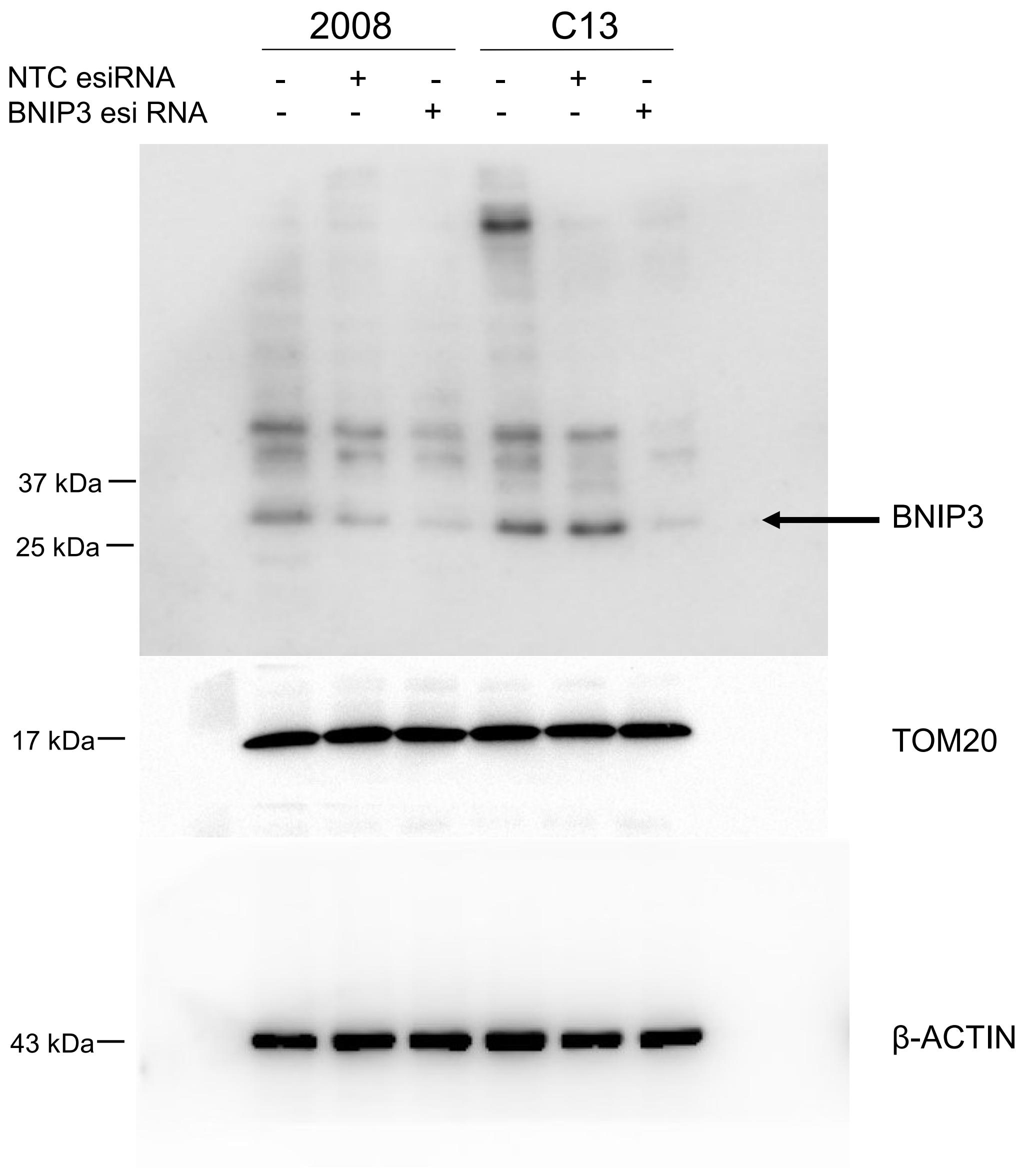

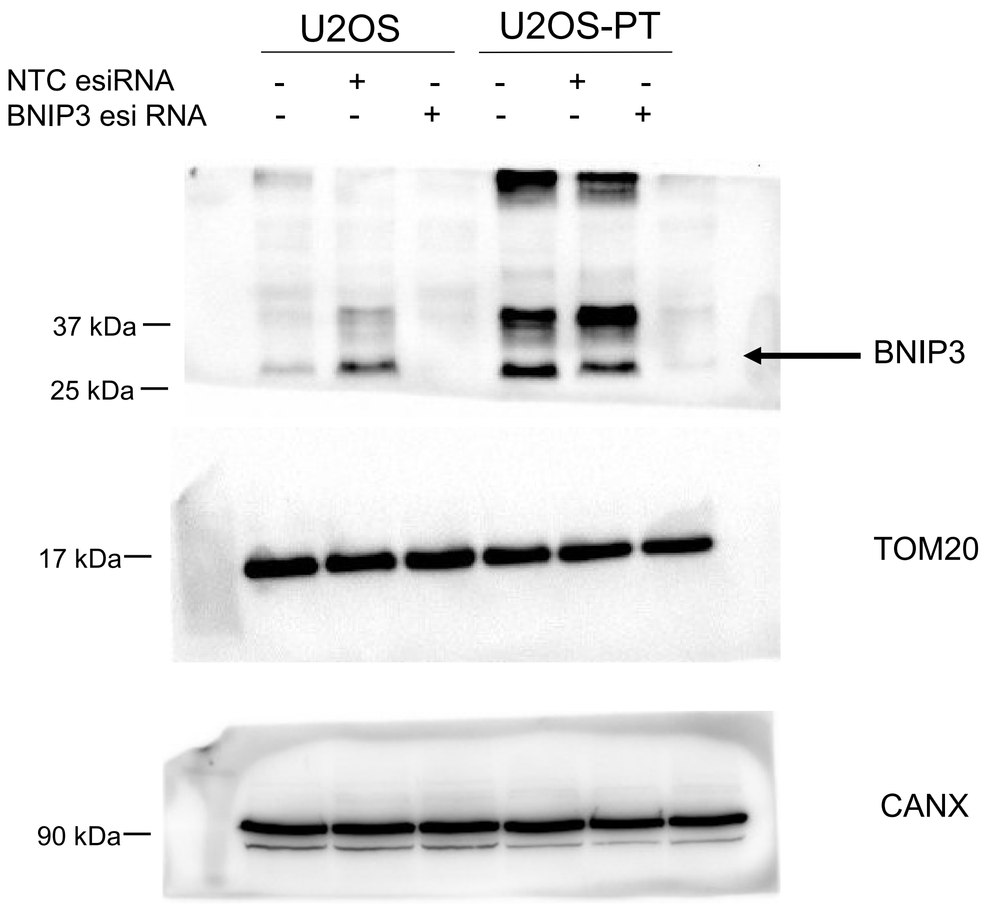

Original uncropped western blots of Figure 4B

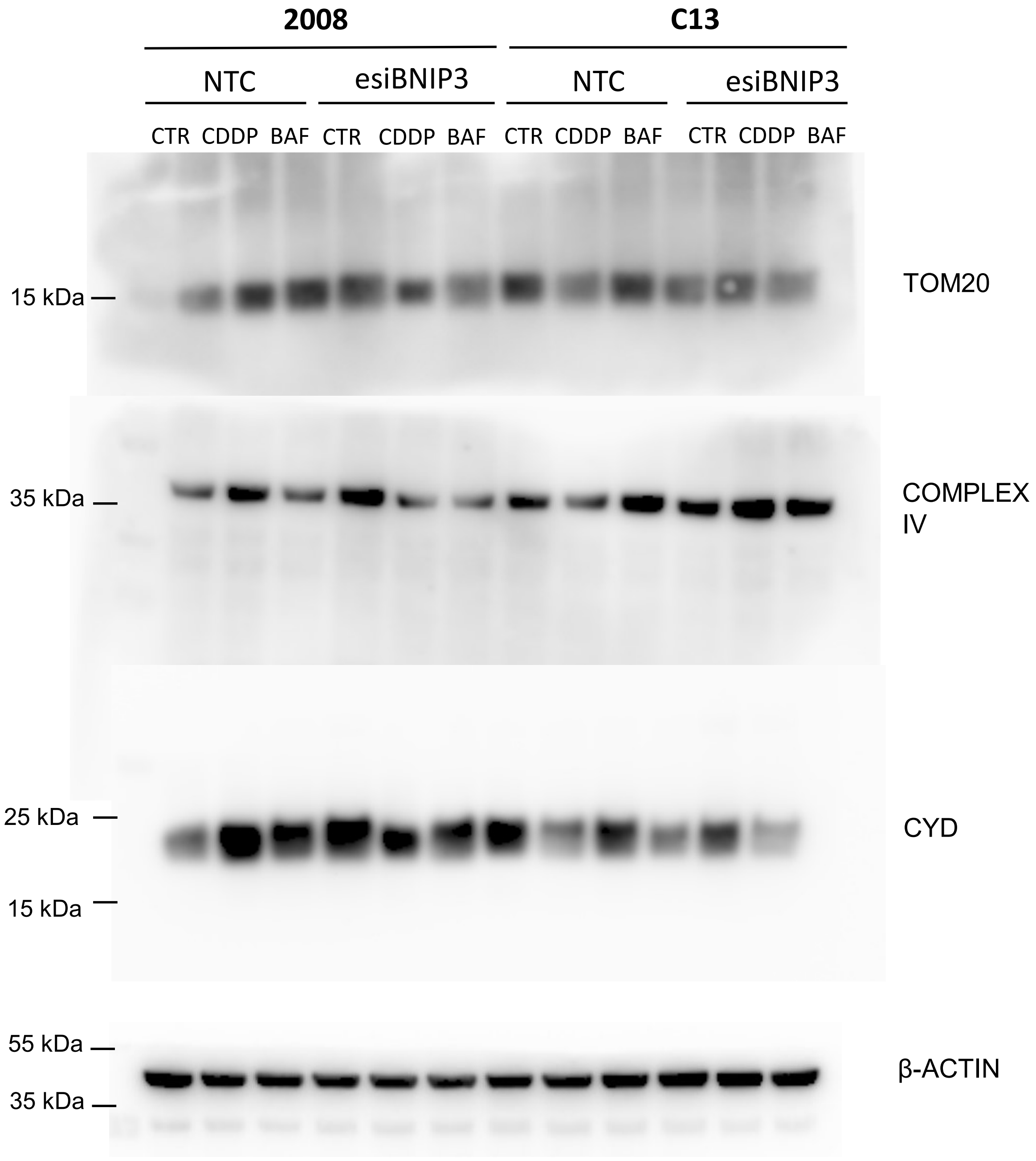

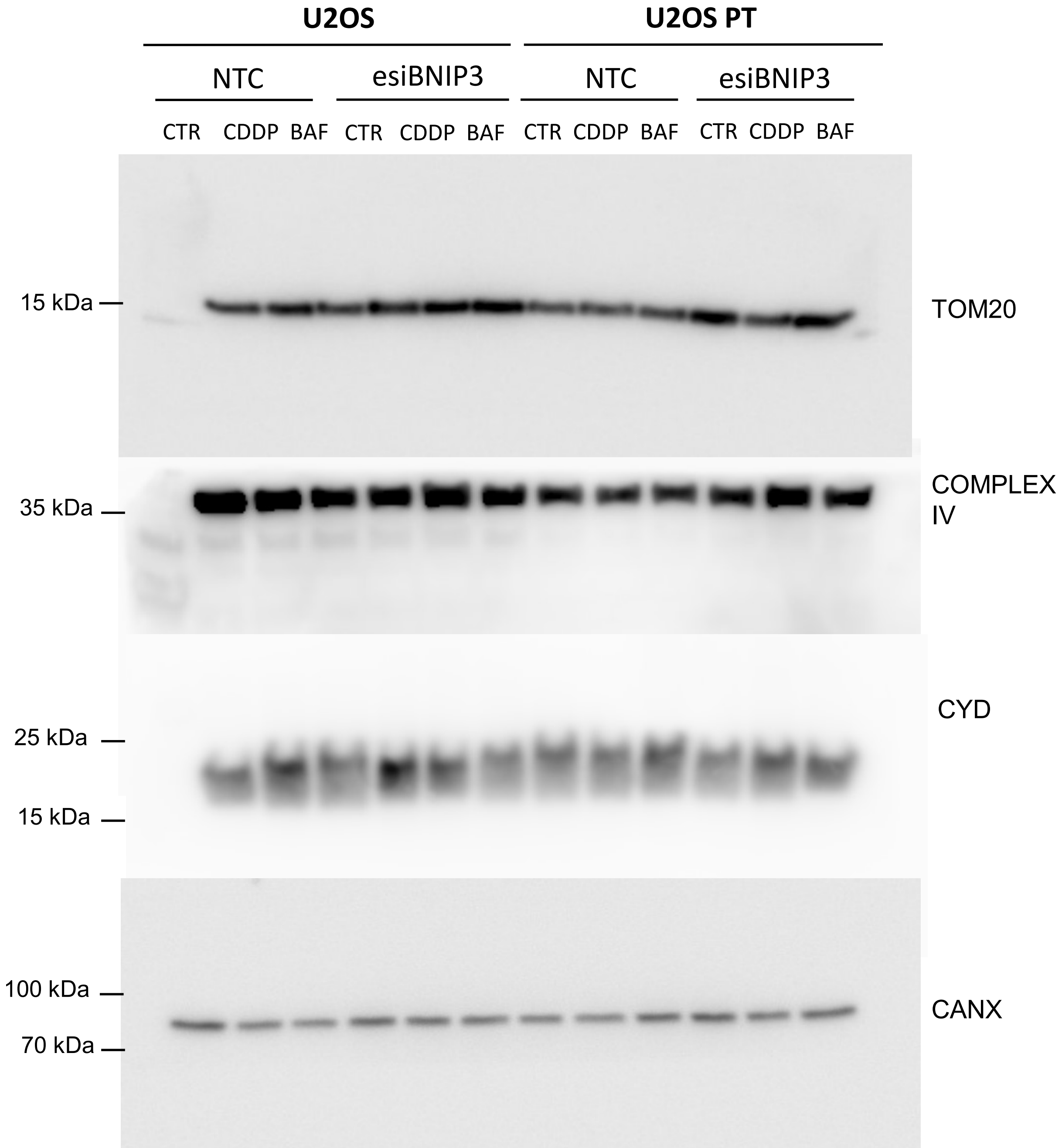

Supplement: Supplementary file 9 — Supplementary Figure 7 [file 41419_2022_4741_MOESM9_ESM.pdf]
